# Loss of qE Does Not Necessarily Lead to Photoinhibition: Sustained Non‐Photochemical Quenching in the Absence of PsbS and Zeaxanthin

**DOI:** 10.1111/pce.70477

**Published:** 2026-03-08

**Authors:** Maximiliano Cainzos, Chen Hu, Maria Dolores Pissolato, Nazeer Fataftah, Sanchali Nanda, Stefan Jansson

**Affiliations:** ^1^ Department of Plant Physiology, Umeå Plant Science Centre Umeå University Umeå Sweden

**Keywords:** high light, new emitting species development, NPQ, photoinhibition, photosynthesis: carbon reactions, photosynthesis: electron transport, sustained quenching

## Abstract

Photosynthetic light‐harvesting complexes mediate light absorption and energy dissipation. By modulating the photosystems' absorption cross‐section, they affect both photosynthetic activity and non‐photochemical quenching (NPQ). These processes are often studied by spectrally integrated chlorophyll fluorescence, masking their associated spectral information. We explore in Aspen and *Arabidopsis* npq mutants how qE affects the development of NPQ spectra under two contrasting conditions: in the absence and the presence of photoinhibition. We introduce a new parameter, the development of new emitting species (NESD), during time‐ and spectrally resolved NPQ inductions, and develop a pipeline to resolve PSII energy‐partitioning heterogeneity. LHCII, PsbS, and zeaxanthin are required for NESD. Combining gas exchange, P700 oxidation, and spectrally resolved kinetics, we show that under photoinhibitory conditions, NES can develop even without PsbS or zeaxanthin, producing sustained quenching independent of photoinhibition of PSII or PSI. Furthermore, the absence of LHCII and CURVATURE THYLAKOID 1 leads to increased photoinhibition, indicating that long‐term photoprotection relies on LHCII and thylakoid plasticity, whereas PsbS and zeaxanthin mainly facilitate LHCII‐dependent quenching. Finally, we show the limitations of traditional parameters in discriminating between photoinhibition and photoprotective sustained quenching and propose time‐resolved monitoring of CO₂ assimilation and Y(II) for their accurate assessment.

## Summary statement

Sustained non‐photochemical quenching at LHCII serves as a photoprotective mechanism independent of PsbS and zeaxanthin, but this protection comes at the cost of reduced light‐use efficiency under fluctuating light conditions. Here, PsbS and zeaxanthin boost performance by enabling proper fine‐tuning of NPQ.

## 1 Introduction

Light harvesting is a fundamental process in green photosynthetic organisms, taking place in photosystem II (PSII) and photosystem I (PSI) in the thylakoid membrane. To harvest and efficiently convert solar energy into chemical energy, photosystems have a matrix of pigments excited by light and transfer excitation energy to reaction centres (RCs) where charge separation takes place (for review, see Croce and van Amerongen 2020). During evolution, chlorophytes have expanded their photosystems by evolving light‐harvesting complexes (LHCs), increasing the absorption cross‐section of PSII and PSI by binding efficiently connected pigments to their RC (Bag 2021). *In planta*, the structure of PSII and PSI core is composed of chlorophylls a and carotenoids, while their LHCs contain chlorophylls a, b and carotenoids. Due to its nature, light is constantly shifting its spectrum and intensity, making the LHC important for the fine‐tuning of energy transfer to its RCs. In addition to their harvesting function, LHCs participate in photoprotection, in which LHCII associated with PSII makes an important contribution (Ruban and Saccon 2022).

Non‐photochemical quenching (NPQ) can be described as the general photoprotective mechanism that reduces the rate of excitation energy transfer to the PSII RC, thereby preventing RC overreduction and ROS production (Ruban 2016). Several NPQ (sub)processes have been described/characterized, from the fast PsbS‐dependent response (qE) to slow ‘sustained quenching’. Since ‘sustained’ refers only to the kinetics, it remains unclear how many distinct molecular mechanisms contribute to this type of quenching. Mechanism(s) dependent on Zeaxanthin (Zea) or lipocalin have been denoted as qZ and qH, respectively (for review, see Malnoë 2018), but direct energy transfer from PSII to PSI has also been shown, in some systems, to provide sustained quenching (see, e.g., Bag et al. 2020). In the absence of qZ, it has been assumed that the slow rise of sustained quenching is solely the product of photoinhibition, which is referred to as qI (Nilkens et al. 2010; Ramakers et al. 2025). Under this assumption, it is expected that increasing sustained quenching should be followed by a strong decline in photochemistry.

Under NPQ conditions, a long‐wavelength fluorescence shoulder (peaking around 720–740 nm, Farooq et al. 2018) is observed when PSII RCs are closed, hereafter referred to as new emitting species (NES). Crucially, this spectral signature has also been linked to photoprotection, whereas its absence is associated with the lack of qE (Holzwarth et al. 2009). Since its discovery in the early 1990s, NES has been attributed to different processes. Originally, it was postulated that qE quenching and LHCII aggregation are the source of the NES and are dependent on PsbS, where Zea enhances this spectral property (Horton et al. 1991; Holzwarth et al. 2009; Johnson and Ruban 2009). Although this signature has been associated with LHCII quenching, it can also be largely affected by PSII‐PSI energy transfer in gymnosperms, where the NPQ spectrum has a strong PSI‐enhanced emission in combination with the contribution of LHCII quenching (Bag et al. 2020). However, recent reports have challenged this interpretation and proposed that the NES originates from PSII closed RCs under actinic light, where dynamic feedback at PSII RCs contributes to photoprotection (Farooq et al. 2018).

One way to address these uncertainties would be to study the NPQ spectral properties of closed PSII RCs in the absence and presence of the PSII antenna. Truncated light‐harvesting antenna (TLA) mutants are therefore an excellent resource for this purpose. Since LHCIIs are not essential for the plant and do not affect the functionality of the RC, several strategies have been developed to study how the photosynthetic machinery behaves in their absence (Cutolo et al. 2023). One strategy is to reduce the physical PSII antenna size by the manipulation of the chlorophyll b content, and chlorophyll a oxygenase (cao) mutants have therefore been used to reduce the LHCII content in plants, as chl b is essential for the stability of several of the LHCs. However, until now, the spectral properties of fluorescence induction and NPQ in TLA plants have not been studied.

In recent years, a large effort has been made to enhance photosynthetic productivity in crops to meet the standards for worldwide food security (Leister 2023). However, photosynthetic improvement has been largely unexplored in trees, which offers the opportunity to boost plant‐based second‐generation biofuels productivity, such as in fast‐growing trees like aspen (Carriquiry et al. 2011). To study some of the key components involved in photosynthetic regulation in aspen, we developed a collection of *cao* mutants, which were combined with our aspen NPQ mutants where PsbS (*psbs*) or Violaxanthin De‐Epoxidase (*vde*) is missing (Nanda et al. 2025). From these lines, we disentangle some of the unknown spectral properties associated with NPQ and assess the contributions of PsbS, Zea, and LHCII to the heterogeneous development of the NPQ spectrum during the initial phase of NPQ. Additionally, we investigate the impact of NPQ on the wavelength‐dependent energy partitioning of PSII, providing new insights into this process. We found different spectral properties for PSII energy partitioning in classical photosynthetic mutants, suggesting a complex interplay between leaf light gradient and NPQ subprocesses. Lastly, by performing photoinhibitory treatments monitored in real‐time by gas exchange and chlorophyll fluorescence, we disentangle some of the components required for photoprotection and their relationship with sustained quenching, photoinhibition and the development of NES in leaves. In addition, we make a comparative analysis using well‐known *Arabidopsis* and barley mutants to determine the extent to which these findings are universal, an aspect that has gained increasing relevance in recent years (Leister 2023).

## 2 Material and Methods

### 2.1 Plant Material

Hybrid aspen T89 (*Populus tremula* × *tremuloides*), barley (*Hordeum vulgare*) and *Arabidopsis* were grown at 22°C/16°C, 16 h/8 h day/night cycle for up to 12 weeks (Figure S1); light intensity was 150–180 μmol photons m^−2^ s^−1^. Aspen npq mutants for PsbS (*psbs*) and Violaxanthin‐De‐Epoxidase (*vde*) were included (Nanda et al. 2025) in combination with *cao* mutants. *Arabidopsis* mutants affecting NPQ key players were used, including *npq1* (lacking VDE), *npq4* (lacking PsbS), L17 (overexpressing PsbS) and *curt1* (CURVATURE THYLAKOID 1 proteins; Havaux and Niyogi 1999; Li et al. 2002; Armbruster et al. 2013). Barley *cao* mutants c107, c109, c102 and c101 were described previously (Bossmann et al. 1997; Mueller et al. 2012).

### 2.2 Design and Cloning of CRISPR‐Single Guide RNAs (sgRNAs) Constructs

Two genes potentially coding CAO were found in the hybrid aspen T89 genome, Potrx053564g16785 (chlorophyll a oxygenase a; CAO1.1) and Potrx056793g18669 (CAO1.2). Potential sgRNAs for target genes were identified with CRISPR‐P (http://cbi.hzau.edu.cn/cgi-bin/CRISPR2/CRISPR), including information regarding off‐target against other genome sequences in the *Populus* genome. The potential off‐targets for sgRNAs were also checked against the hybrid aspen T89 genome using the BLAST method on the PlantGenIE website (https://plantgenie.org/BLAST). sgRNA1 (GTGGAAGAAGGAGTTGCCACG) and sgRNA2 (GGATCCTCAACATCAAAGAG) were designed to target CAO1.1; sgRNA3 (GTAAGATGCCACAGTTTAAA) and sgRNA4 (GACCTTGGTTCAGTGAATGA) to target CAO1.1 and CAO1.2 together (mentioned here as CAO2). sgRNAs were introduced into entry vectors by site‐directed mutagenesis PCR. GreenGate entry and destination vectors were acquired from Addgene. The final vector (containing promoter, Cas9 CDS, terminator, two sgRNAs and resistance cassette) was assembled by GreenGate reaction as described in André et al. (2022). *Escherichia coli* strain DH5α was used for amplification of all plasmids, which were then confirmed by sequencing (Eurofins). Vectors with different combinations of sgRNAs were transformed into Hybrid aspen T89 using a standard protocol. At least 30 individual transgenic lines from each transformation were screened for target gene deletions or SNPs using PCR and confirmed by sequencing. Forward: 5′‐ACTTGCAAGCTAATCACACC‐3′ and reverse: 5′‐CAAGGTGAGACTTGAAAGTC‐3′ primers were used to genotype the transformation CAO1; forward: 5′‐TGTGATCGGTGTGTGATTTTC‐3′ and reverse: 5′‐TATCACCAAATTTTCAATAC‐3′ were used to genotype the CAO1 gene in transformation CAO2; forward: 5′‐ATAGTTTCCTGCTAAGGCTA‐3′ and reverse: 5′‐TCCCAGCCTGATGTTTATTG‐3′ were used to genotype CAO1.2 in transformation CAO2. CRISPR of CAO1 transformation caused deletion lines, while CAO2 introduced stop coding in the sgRNA3 location in both CAO1 and CAO2 genes, producing a fragment of 67 amino acids instead of the expected full CAO that formed from around 535 amino acids.

### 2.3 Pigment Extractions

Absorption spectra of leaves and thylakoid extractions in 80% acetone were recorded by a UV‐Vis spectrophotometer, Shimadzu UV‐2600i. The total amounts of chlorophylls (a + b) and carotenoids were obtained by fitting the spectra, as previously described by Croce et al. (2002). From these, ratios of chlorophyll a/b and chlorophyll/area were calculated.

### 2.4 Thylakoid Membrane Isolation and Biochemical Analysis

Thylakoids were prepared as described by Caffarri et al. (2009) from overnight dark‐adapted plants. BN‐PAGE was performed on 4%–12%‐gradient (Järvi et al. 2011). Eight micrograms of chlorophylls were solubilized in a final detergent concentration of 2% β‐DM and then loaded in each lane. 2D separation was performed from first dimension strips native‐PAGE as described by Järvi et al. (2011). After electrophoresis, the proteins were visualized by Coomassie Blue staining.

SDS‐Page was performed as described in He (2011). Thylakoid solubilization was performed for 2 h at 37°C to avoid protein membrane aggregation. Gels were transferred for 20 min to a nitrocellulose membrane and blocked in TBS‐T milk 5% for 1 h at RT. Blot was incubated overnight at 4°C with primaries antibodies for: CP43 (AS11‐1787), Lhcb1(AS01‐004), Lhcb2(AS01‐003), Lhcb3(AS01‐002), Lhcb4(AS04‐045), Lhcb5(AS01‐009), Lhcb6(AS01‐010), Lhca1(AS01‐005), Lhca2(AS01‐006), Lhca3(AS01‐007), Lhca4 (AS01‐008, ATPC (AS08‐312), PetA (AS20‐4377), PsaA (AS06‐172) and PsbS (AS09‐533). Membranes were washed three times for 5 min with TBS‐T at RT and incubated with the secondary antibody (AS09‐602) for 1 h at RT. Membranes were washed and developed with Clarity (Bio‐Rad) and imaged by Azure 600.

### 2.5 TEM Analysis

Slices from the same developmental age of aspens T89, *cao1.11* and *cao2.18* leaves from the light‐adapted state were cut in distilled water and fixed in 4% paraformaldehyde and 2.5% glutaraldehyde in 0.1 M sodium cacodylate buffer, pH 7.4. Samples were rinsed with sodium cacodylate buffer and incubated in 1% osmium tetroxide. Fixed material was dehydrated in increasing concentrations of ethanol and eventually embedded in Spurr resin. Seventy‐nanometer ultrathin sections were made with a diamond knife and contrasted in uranyl acetate and citrate. Images were obtained with a Talos 120 C electron microscope operating at 120 kV in combination with a Ceta 16 M CCD camera. ImageJ software was used to count thylakoid stacks per grana and chloroplast by a blind analysis. A total of 20 chloroplasts was counted per line: 411 stacks for T89, 388 stacks for cao1 and 439 stacks for *cao2*.

### 2.6 Integrated Chlorophyll Fluorescence by PAM Fluorometry

Chlorophyll fluorescence was measured by a Dual PAM‐100 (Walz). Plants were dark‐adapted for 1 h before measurements. Saturating pulse (SP) of 10 000 μmol photons m^−2^ s^−1^ of 500 ms was applied to estimate the maximal fluorescence in the dark‐adapted (F_m_) and light‐adapted state (F′_m_). Induction of NPQ was achieved by an actinic light of 1000 μmol photons m^−2^ s^−1^. To record the NPQ kinetics, a SP was applied for 20 s for 8 min of actinic light. After the actinic light was turned off, SP was applied after 20 s, followed by 10 SP with a delay of 1 min.

We estimated NPQ from chlorophyll quenching fluorescence by the saturating light pulse method, where NPQ as a function of time can be described as:

(1) $$\mathrm{NPQ}=\frac{F_{m}\left(t_{0}\right)}{F_{m}\left(t\right)}-1.$$

In the equation above, F_m_ (t_0_) is the yield of maximal fluorescence in a dark‐adapted state, or F_m_, and F_m_ (t) is the maximal fluorescence measured at any time during the NPQ kinetic, also referred to as F′_m_ (Baker 2008). Equation (1) is a simplified description of the integrated chlorophyll fluorescence intensity measured by PAM fluorometry (applies for instruments like Dual‐PAM‐100, Handy PEA+, JTS‐100 and Licor‐6800, among others), which can be formally expressed as an integral of fluorescence as a function of wavelength:

(2) $$\mathrm{NPQ}=\frac{F_{m}\left(t_{0}\right)}{F_{m}\left(t\right)}-1=\frac{\int_{\lambda_{1}}^{\lambda_{2}}F_{m}\left(\lambda ,t_{0}\right)\;d\lambda }{\int_{\lambda_{1}}^{\lambda_{2}}F_{m}\left(\lambda ,t\right)\;d\lambda }-1.$$

In Equation (2), *λ*_1_ and *λ*_2_ are the wavelengths where the long‐pass filter of PAM fluorometry integrates the fluorescence intensity. In leaves, self‐reabsorption can mislead spectral data interpretation. To avoid that, F_m_(t) can be corrected by the reflected and transmitted radiation using the correction factor ‘*f*’ which is *λ* dependent (Chukhutsina et al. 2019):

(3) $$\int_{\lambda_{1}}^{\lambda_{2}}F_{m}\left(\lambda ,t\right)\;x\;f\left(\lambda \right)\;d\lambda ,$$

where ‘*f* ’:

(4) $$f=\frac{\ln\frac{1}{\left(r_{\lambda e}+t_{\lambda e}\right)}+\ln\;\frac{1}{\left(r_{\lambda }+t_{\lambda }\right)}}{n\frac{1}{r_{\lambda e}+t_{\lambda e}}}\times \frac{1-r_{\lambda e}-t_{\lambda e}}{1-(r_{\lambda e}+t_{\lambda e})(r_{\lambda e}+t_{\lambda e})}.$$

Here, *r* and *t* denote the reflected and transmitted radiation, respectively, while *λe* refers to the excitation wavelength and *λ* to the detection wavelength. As long as the self‐reabsorption properties remain unchanged, for example during a NPQ induction using red‐actinic light (Wilson and Ruban 2020), NPQ corrected would be calculated as:

(5) $$\mathrm{NPQ}\;\mathrm{corr}=\frac{\int_{\lambda_{1}}^{\lambda_{2}}F_{m}\left(\lambda ,t_{0}\right)\times f\left(\lambda \right)\;d\lambda }{\int_{\lambda_{1}}^{\lambda_{2}}F_{m}\left(\lambda ,t\right)\times f\left(\lambda \right)\;d\lambda }-1=\frac{\int_{\lambda_{1}}^{\lambda_{2}}F_{m}\left(\lambda ,t_{0}\right)\;\;d\lambda }{\int_{\lambda_{1}}^{\lambda_{2}}F_{m}\left(\lambda ,t\right)\;d\lambda }-1=\frac{F_{m}\left(t_{0}\right)}{F_{m}\left(t\right)}-1=\mathrm{NPQ}.$$

Therefore, any optical changes due to leaf structural changes would cancel out, and NPQ will reflect only changes in chlorophyll fluorescence yields. This underlying assumption has allowed the photosynthetic community to study photosynthesis by PAM fluorometry (Schreiber et al. 1986). Note that the same assumptions are applied to the well‐known parameters used to study energy partitioning or the redox state of the PSII RC (Baker 2008), where:

(6) $$Y\left(\text{II}\right)=\frac{F_{m}\prime -F^{\prime }}{F_{m}\prime }=1-\frac{F^{\prime }}{F_{m}\prime }=1-\frac{\int_{\lambda_{1}}^{\lambda_{2}}F\left(\lambda ,t\right)\times f\left(\lambda \right)d\lambda }{\int_{\lambda_{1}}^{\lambda_{2}}F_{m}\left(\lambda ,t\right)\times f\left(\lambda \right)\;d\lambda }=Y\left(\mathrm{II}\right)\;\mathrm{corr},$$

(7) $$Y\left(\text{NO}\right)=\frac{F^{\prime }}{F_{m}}=\frac{\int_{\lambda_{1}}^{\lambda_{2}}F\left(\lambda ,t\right)\times f\left(\lambda \right)d\lambda }{\int_{\lambda_{1}}^{\lambda_{2}}F_{m}\left(\lambda ,0\right)\times f\left(\lambda \right)\;d\lambda }=Y\left(\mathrm{NO}\right)\;\mathrm{corr},$$

(8) Y(NPQ)=1−Y(II)−Y(NO),

(9) $$\text{qP}\;=\frac{F_{m}\prime -F^{\prime }}{F_{m}\prime -F_{o}\prime }=\frac{\int_{\lambda_{1}}^{\lambda_{2}}F_{m}\prime \left(\lambda ,t\right)\times f\left(\lambda \right)\;d\lambda -\int_{\lambda_{1}}^{\lambda_{2}}F^{\prime }\left(\lambda ,t\right)\times f\left(\lambda \right)d\lambda \;}{\int_{\lambda_{1}}^{\lambda_{2}}F_{m}\prime \left(\lambda ,t\right)\times f\left(\lambda \right)\;d\lambda -\int_{\lambda_{1}}^{\lambda_{2}}F_{o}\prime \left(\lambda ,t\right)\times f\left(\lambda \right)d\lambda \;}=\mathrm{qP}\;\mathrm{corr}.$$

Here, F′ and Fo′ stand for the minimal yield of fluorescence at any given time during a NPQ kinetic and the minimal yield of fluorescence once all the RCs have been reopened, respectively. To determine Fo′, we used the approximation Fo′ = Fo/(Fv/Fm + Fo/Fm′) as described by Oxborough and Baker (1997).

### 2.7 Wavelength‐Dependent NPQ Inductions

Fast fluorescence inductions were recorded in dark‐adapted leaves at 686, 700 and 730 nm using a ChloroSpec L1, by three fast photodiode channels equipped with 10 nm FWHM filters (Nanda et al. 2024). Fo was set at 12.6 μs and the STF (single turnover flash) duration to 130 μs and an intensity of 60 000 μmol photons m^−2^ s^−1^. STF burst duration was 0.54 ms. A MTF (Multi‐Turnover Flash) of 500 μs and an intensity of 15 000 photons μmol m^−2^ s^−1^ was applied subsequently. Values coming from the MTF were used to estimate the NPQ evolution spectrum, by full spectral detection by an optical spectrometer (Nanda et al. 2024).

To perform short NPQ kinetics, 1000 μmol photons m^−2^ s^−1^ were applied for a range of 8 min. A logarithmic sequence was applied to obtain MTF(*λ*,t), where each FI + MTF was spaced by 1, 1, 1, 1, 2, 3, 6, 12, 23, 30, 44, 65, 100 and 191 s. Subsequently, dark recovery was measured for 10 min applying a FI + MTF spaced by 5, 10, 20, 40, 45, 60, 60, 60, 60, 60, 60, 60 and 60 s. Long NPQ kinetics were done at 1500 photons m^−2^ s^−1^ for 4572 s followed by 1200 s of dark relaxation. Plants were dark‐adapted for 1 h before measurements.

Here, we studied NPQ spectral kinetics using red‐actinic light and followed the changes of the maximal fluorescence emission spectrum over time, F_m_(*λ*,t), coming from the MTF. In this analysis, NPQ (*λ*,t) can be described as:

(10) $$\mathrm{NPQ}\;(\lambda ,\;t)=\frac{F_{m}\left(\lambda ,t_{0}\right)}{F_{m}\left(\lambda ,t\right)}-1.$$

F_m_ (*λ*,t_0_) is the fluorescence emission spectrum of a dark‐adapted sample, and F_m_ (*λ*,t) is the fluorescence emission spectrum at any time during the NPQ kinetics. Note that Equation (10) is identical to Equation (1) and can be corrected by the correction factor ‘*f*’ for self‐reabsorption:

(11) $$F_{m}\left(\lambda ,t\right)\times f\;\left(\lambda \right).$$

Therefore, it can be deduced that:

(12) $$\mathrm{NPQ}\;\mathrm{corr}\;(\lambda ,t)=\frac{F_{m}\left(\lambda ,t_{0}\right)\;x\;f\left(\lambda \right)}{F_{m}\left(\lambda ,t\right)\;x\;f\left(\lambda \right)}-1=\frac{F_{m}\left(\lambda ,t_{0}\right)\;}{F_{m}\left(\lambda ,t\right)}-1=\mathrm{NPQ}\;(\lambda ,\;t).$$

As above, any optical changes due to leaf structural changes would cancel out and NPQ (*λ*,t) will reflect only changes in chlorophyll fluorescence yields. As we induce NPQ by red‐actinic light, it is highly unlikely that self‐reabsorption changes during these experiments; hence, spectra need not be corrected, an assumption also made in PAM fluorometry. To follow the dynamics of NES emergence during NPQ, we developed the parameter NES development (NESD).

(13) NESD=NPQ(685,t)−NPQ(λ,t).

Here, NESD captures the spectral heterogeneity of NPQ, thus the deviation of NPQ at any wavelength *λ* relative to the reference 685 nm, where PSII fluorescence dominates.

Y(II) (*λ*,t), Y(NO) (*λ*,t) and Y(NPQ) (*λ*,t) were calculated according to Equations ((6), (7), (8)), respectively, from each fast fluorescence induction detected at 686, 700 and 730 nm. From them, PSII energy partitioning heterogeneity was determined for Y(II), Y(NO) and Y(NPQ) by the following equations:

(14) Y(II)(686,t)−Y(II)(700,t),

(15) Y(II)(686,t)−Y(II)(730,t),

(16) Y(NPQ)(686,t)−Y(NPQ)(700,t),

(17) Y(NPQ)(686,t)−Y(NPQ)(730,t),

(18) Y(NO)(686,t)−Y(NO)(700,t),

(19) Y(NO)(686,t)−Y(NO)(730,t).

From these differences, dimensional reduction was applied by clustering analysis in combination with PCA.

### 2.8 Integrated Chlorophyll Fluorescence and Gas Exchange

Net CO_2_ assimilation (A), stomatal conductance (gsw), intercellular CO_2_ (Ci) and chlorophyll a fluorescence were measured using an infrared gas analyzer (model LI‐6800, LI‐COR, Lincoln, NE, USA) in a 2 cm^2^ cuvette at 1500 μmol photons m^−2^ s^−1^ (Tyystjärvi and Aro 1996; Munekage et al. 2002; Nawrocki et al. 2021). During measurements, leaf temperature was maintained at 25°C and humidity at 60%, while the gas mixture contained 21% O₂ and 400 ppm CO₂. The same conditions were applied to all measurements performed. Logs were performed every 2 min by the auto‐gen loop function in a light interval of 4680 s followed by a dark interval of at least 1200 s. Each kinetic has a duration of at least 1:50 h. Sustained quenching effect was monitored by replacing the dark interval with a fluctuating light response of 1200 s, with 150 and 1500 μmol photons m^−2^ s^−1^ light steps of 1 min duration. Log was performed every minute in this protocol.

A pre‐dark phase of 240 s before each induction was done to stabilize the samples and measure Fm. NPQ, Y(II), Y(NO), and qP were determined as mentioned above.

To estimate the effect of photoinhibition on the overall productivity during the light treatment, we normalized A to the maximal A value obtained during the kinetic, denoted as A_(norm)_. Thus, a decline in A_(norm)_ at any given time after the system reached A_max_ is a proxy of photoinhibition. To measure the extent of photoinhibition by traditional parameters, we calculated qI (Li et al. 2019; Smith et al. 2024), at the end of the dark‐relaxation phase:

(20) qI=(Fm−F″m)/F″m.

We also determined photoinhibition by the traditional parameter Fv/Fm (Nawrocki et al. 2021). Alternatively, we monitored changes in the light‐adapted state by measuring Y(II) norm., which is the normalized value of Y(II) to the maximal Y(II) value obtained during the treatment.

Chlorophyll fluorescence and P700 redox changes were monitored using a Dual‐PAM 100 (FIBER version). Measurements were done as described in Section 2.6 with the modification of including the dual mode for P700 detection. Pm was determined in each SP. After dark‐adaptation, the first SP was used to estimate Fv/Fm. A strong far‐red light (FRL) pulse (250 photons m^−2^ s^−1^; max intensity) of 15 s and a subsequent SP were applied to fully oxidize P700 and obtain Pm. During the photoinhibitory treatment, SP was applied every minute, and Y(ND) was determined in combination with Y(II). Y(ND) was estimated as:

(21) Y(ND)=P700ox/Pm.

After 80 min of high‐light (HL) treatment and a dark relaxation phase of 20 min, Pm′ was measured by a SP and a strong FRL pulse + SP. From there, photoinhibition was calculated as:

(22) PSIphotoinhibition=Pm′/Pm.

### 2.9 Statistical Analysis

Statistical analysis was performed in RStudio through an in‐house pipeline to analyze the raw data signals from ChloroSpec and Dual‐PAM‐100 from SP kinetics and processed data from Licor‐6800. To perform and plot the results from linear regression, PCA, cluster analysis, ANOVA and Tukey test, the libraries ggplot2, stats, cluster, factoextra, multcomp and multcompview were used. For clustering, supervised analysis was performed, where the optimal number of clusters was determined based on the elbow method.

## 3 Results

### 3.1 *Aspen cao* Mutants Have a Reduced Physical PSII Antenna Size and NPQ

**Figure 1:**
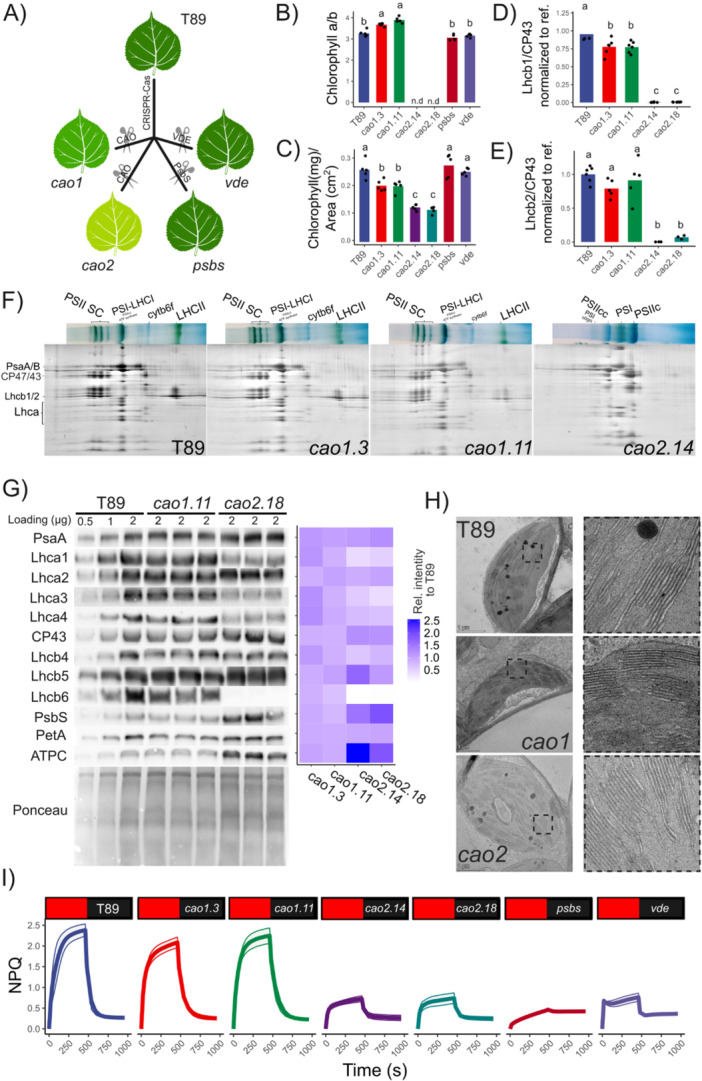
LHCII, PsbS and zeaxanthin modulate NPQ in aspens. (A) Schematic describing chlorophyll a oxygenase (CAO), PsbS and VDE mutants production by CRISPR‐Cas9 in aspen (T89). (B) and (C) Chlorophyll a/b and chlorophyll content per leaf from T89, *cao1*, *cao2*, *psbs* and *vde* mutants. Pigment content values are means from five biologically independent experiments; each point is from one replicate. Shared letters indicate non‐significant differences between the groups (Tukey's tests, *p* < 0.05). Note that *cao* mutants *cao2.14* and *cao2.18* lack chlorophyll b (n.d). (D) and (E) Relative signal of Lhcb1 and Lhcb2 to CP43 were quantified by immunoblotting and normalized to T89. Shared letters indicate non‐significant differences between the groups (Tukey's tests, *p* < 0.05). (F) 2‐D BN/SDS‐PAGE from aspen thylakoids solubilized in 2% β‐DM. Eight micrograms of chlorophylls were loaded per lane. In Figures S1 and S2, BN‐Page and blots are shown. (G) Immunoblot analyses of thylakoid proteins from T89, *cao1* and *cao2* using specific antibodies for PSI, PSII proteins, PsbS, ATP synthase (ATPC) and cytb6f (PetA) complex. 0.5 to 2 μg of total Chl were loaded into each well. At the right, heat map of thylakoid protein content relative to signal from T89 (100%). Gradient from white (0%), to lilac (100%), to blue (250%) reflects increasing relative levels when compared to T89. In Figure S3, blots are shown for the analyzed lines. (H) T89, *cao1.11* and *cao2.18* electron micrographs of representative chloroplasts. This experiment was repeated on two biologically independent samples with similar results. (I) NPQ inductions performed at 1000 μmol photons m^−2^ s^−1^ in the collection of NPQ aspen mutants using a standard pulse amplitude modulated fluorometer (Dual‐PAM‐100). On top of each trace is indicated the corresponding genotype. The induction and relaxation phases are represented by the red and black boxes, respectively. Data are mean ± SD (*n* = 5 biologically independent experiments). In Figure S4, extended data from Figure 1 is shown.

To study how LHCs and major regulators affect NPQ and the development of NES in photoinhibitory and non‐photoinhibitory conditions in *Poplar*, we generated a collection of aspen *cao* mutants using CRISPR‐CAS9, in combination with PsbS (*psbs*) and Violaxanthin de‐epoxidase (*vde*) mutants (Nanda et al. 2025; Figure 1). We identified two copies of the CAO gene, targeted one (*cao1*) or both (*cao2*) to partially or completely disrupt the synthesis of chlorophyll b (Figures 1 and S1). This resulted in a set of aspen lines with varying CAO activity and, consequently, different chlorophyll b content. An increase in chlorophyll a/b ratio was associated with a decrease in the chlorophyll content per unit leaf area (Figure 1B,C). In *psbs* and *vde*, chlorophyll a/b ratios and chlorophyll content were the same as in the wild type (T89; Figure 1B,C). As expected, changes in chlorophyll b content in *cao* were reflected in a reduction of the PSII physical antenna size (Figures 1D–G and S1–S3). The lines *cao1.3* and *cao1.11* showed only small changes in their chlorophyll a/b ratio and thylakoid composition, whereas the absence of chlorophyll b (*cao2.14* and *cao2.18*) compromised LHCB accumulation and PSII supercomplex stability. Unlike *Arabidopsis ch1*, in which only Lhcb5 is stable (Havaux et al. 2007; Kim et al. 2009), aspen retained both Lhcb4 and Lhcb5 in the absence of chlorophyll b. Although *cao* mutants are generally thought to lack Lhcb proteins, comparative analyses between Lhcb knockout mutants and *ch1* from *Arabidopsis* reveal significant differences, suggesting that minor antenna proteins may still be present in the absence of chlorophyll b (Guardini et al. 2025).

We also examined the accumulation of core subunits of the four photosynthetic complexes, as well as PsbS (Figure 1F,G). Overall, *cao1* was characterized by an increased physical absorption cross‐section of PSI (~105%–114%), reflected in higher levels of Lhca1–4 and PsaA, along with a slight reduction in CP43 (~86%–90%) and Lhcb proteins compared to T89. In *cao2*, we observed a similar increase in PsaA (~115%–120%) and CP43 (~120%–125%), but the stability of the PSI antenna was impaired, although Lhca2 remained largely stable (~90%–100%). Regarding ATP synthase and the cytochrome b6f complex, ATPC accumulated to 200% in *cao2* but was unchanged in cao1 (~92%–101%), while PetA levels remained unchanged in *cao2* (~97%–99%) and reduced in *cao1* (~90%–93%). Finally, PsbS levels were increased by 50% to 90% in *cao2* and reduced by 83%–90% in *cao1* in agreement with the reduction of PSII RCs. Given that LHCII plays a role in grana stacking, we quantified grana stacking in the *cao1.11* and *cao2.18* lines and observed no differences between them (Figures 1H and S4). Here, the presence of Lhcb4 and Lhcb5 in *cao2* may play a compensatory role in thylakoid stacking, as proposed for Lhcb5 in *ch1* (Guardini et al. 2022).

NPQ in Aspen was dependent on PsbS, Zea and LHCII (Figure 1I). *Cao1* showed NPQ induction patterns like T89, but NPQ was strongly reduced in *cao2* lines, as expected for mutants lacking LHCII (Figure 1G,H). Additionally, both *psbs* and *vde* showed a marked reduction in NPQ, reflecting the absence of qE and qZ components, respectively. When NPQ was normalized (Figure S4), we observed the rapid induction of the qE‐associated component in T89, *cao1*, *cao2*, and *vde*, but not in *psbs*, as expected. The onset of qE was noticeably faster in both *cao1* and *cao2* compared with T89. Therefore, the fast inductions of qE in *cao2* suggest the action of PsbS and Zea components independent of LHCII (Nicol et al. 2019; Guardini et al. 2025).

### 3.2 NPQ Evolution Spectrum Reflects Changes in Chlorophyll Fluorescence Yields in Leaves

A limitation of PAM fluorometry is that the fluorescence signal is integrated above a certain detection cutoff wavelength (710 nm); hence, potential variations in the short‐wave emission spectrum, where PSII contribution dominates, are ignored (Pfündel 2021). To break the bidimensional limit of NPQ, it is necessary to time‐resolve the fluorescence emission spectra during light inductions (Nanda et al. 2024). We performed NPQ spectral kinetics using red‐actinic light and monitored the changes of the maximal fluorescence emission spectrum over time, Fm (*λ*,t), by full spectral detection with an optical spectrometer. We derive NPQ (*λ*,t) as described in Equation (10), where self‐reabsorption cancels out, like in PAM fluorometry (Equation 5). Therefore, NPQ (*λ*,t) and NPQ reflect changes in chlorophyll fluorescence yields at any given time.

**Figure 2:**
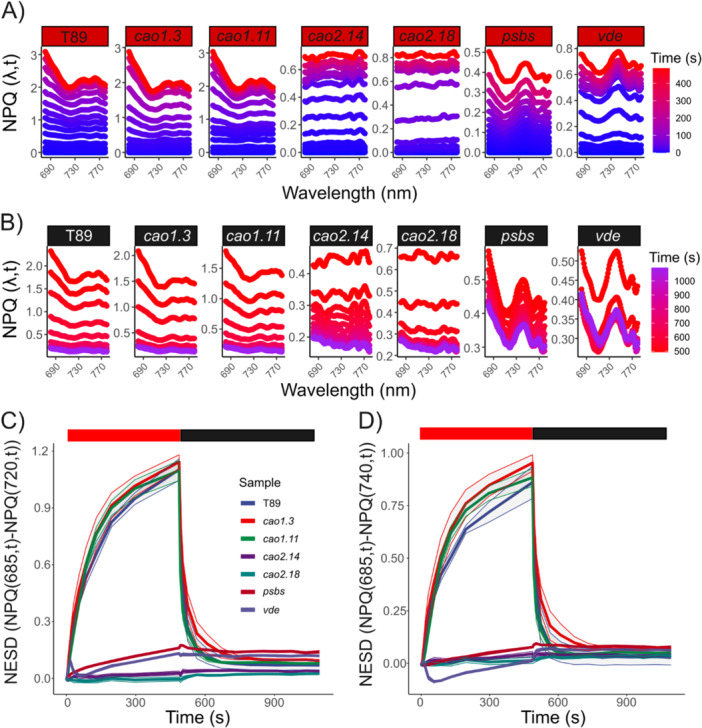
LHCII, PsbS and zeaxanthin are required for the development of new emitting species under non‐photoinhibitory conditions. NPQ inductions were performed at 1000 μmol photons m^−2^ s^−1^ in the collection of NPQ aspen mutants. Induction and relaxation phase NPQ evolution spectra are shown in (A) and (B), respectively. Each NPQ evolution spectrum represents one biological replica. Full spectral detection was performed by an optical spectrometer (range 680–780 nm). New emitting species development (NESD) was calculated from NPQ evolution spectra as (NPQ_(685,t)_ − NPQ_(*λ*,t)_), where *λ* = 720 nm in (C) and *λ* = 740 nm in (D). Data are mean ± SD (*n* = 5 biologically independent experiments). [Color figure can be viewed at wileyonlinelibrary.com]

We found that NPQ (*λ*,t) depends on PsbS, Zea and the LHC antenna (Figures 2 and S5–S8). In T89 and *cao1*, NPQ spectra showed canonical behaviour, like other higher plants (Nanda et al. 2024). However, differences in the NPQ spectra were absent in *cao2* and reduced in *psbs* and *vde*. To quantitatively estimate NPQ (*λ*,t) changes, we introduce a new coefficient named NESD, described in Equation (13). In these specific conditions, we compared NPQ (685,t) with NPQ (720,t), where NES is larger in T89 (Figure 2C). In T89 and *cao1*, the differences between NPQ (685,t) and NPQ (720,t) were pronounced (NESD = 1 at the end of the NPQ induction), with NESD following the dynamics of classic NPQ inductions and relaxations. In contrast, we found that NESD tends to 0 in *cao2*, whereas in *psbs* and *vde* was reduced to 0.1. Thus, during NPQ, the presence of LHCII, PsbS and Zea is necessary for the development of NES. However, when comparing other wavelengths such as NPQ (740,t), we observed negative values for NESD in *vde*, indicating that NPQ was higher at longer detection wavelengths, contrary to what is typically expected (Figure 2D). Moreover, NESD was highly dynamic: following induction during the light phase, it relaxed by up to 75% within 16 s and was completely abolished after 60 s (Figure S9).

Because NESD influences the overall estimation of NPQ, particularly at longer detection wavelengths, we compared NPQ(*λ*,t) at 685, 720 and 740 nm with NPQ integrated. Here, NPQ integrated corresponds to the technical approach used in chlorophyll fluorometers equipped with long‐pass filters (Figures S8, S10 and S11). This analysis revealed that, relative to the reference lines, the NPQ values of npq and *cao2* mutants appeared up to 50% higher when measured at shorter detection wavelengths. Moreover, we observed that NPQ at 720 nm is 10% smaller when compared to integrated NPQ, mainly a product of NESD peaking at that wavelength (Figure 2F). In Figures S9–S11, we explained in more detail some of the consequences of these observations. We also compared NPQ values obtained with the DUAL‐PAM‐100 to those obtained from the integrated Chlorospec method in the same plants from similar developmental leaves. When the two approaches are compared, the integrated Chlorospec measurement yields NPQ values that are approximately 10% lower, reflecting the reduced influence of the light gradient in the DUAL‐PAM‐100 (Figure S11). However, when NPQ values from the DUAL‐PAM‐100 are compared specifically with the 686 nm detection from Chlorospec, the PAM integrated method underestimates NPQ by 30%.

### 3.3 PSII Energy Partitioning Heterogeneity Emerges From the Interaction of the Leaf Light Gradient and NPQ Regulation

PSII energy partitioning is a valuable tool for understanding the fate of excitons in photosynthesis. The pioneering work of Genty et al. (1989) directly linked chlorophyll a fluorescence to photosynthesis, introducing the parameter Y(II), and later Y(NO) and Y(NPQ) were derived (Baker 2008; Klughammer and Schreiber 2008). These parameters are typically measured under red‐actinic light conditions, where the self‐reabsorption effect is cancelled out (see above, Equations (6), (7), (8)); therefore, they do not require correction for reabsorption in PAM fluorometry analyses, nor in our wavelength‐dependent fluorescence method. In Figure S12, results from PAM fluorometry are shown. Upon NPQ induction (1000 μmol photons m^−2^ s^−1^; Figure 1), once the system reached steady state in T89, Y(NPQ) dominates, followed by Y(NO), which constrains the use of energy for photosynthesis by PSII, represented by Y(II), where only 5%–6% of absorbed photons are used for charge separation. *Cao1* showed a similar trend, but in the absence of qE or LHCII, however, the system was mainly dominated by Y(NO), which was up to twice as high as in T89.

**Figure 3:**
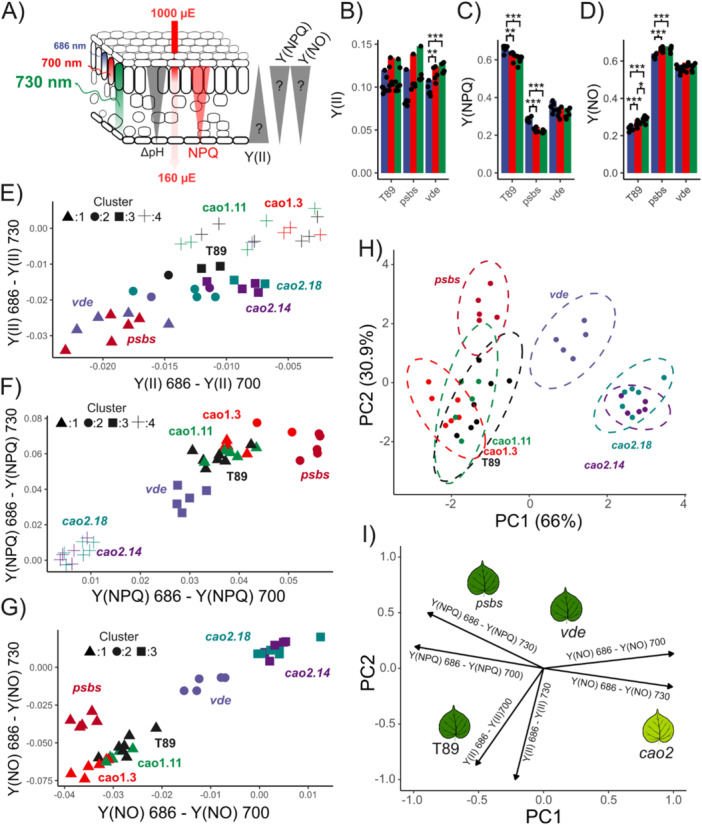
PSII energy partitioning heterogeneity upon NPQ is mutant dependent. (A) A schematic illustrating the relationships between monochromatic excitation through the leaf blade, known as the light gradient (depicted by the red arrow), and the resulting NPQ/ΔpH changes, represented by red and grey triangles, respectively. It is expected that, under monochromatic excitation, PSII excitation will be greater on the abaxial side of the leaf compared to the adaxial side, leading to larger stromal‐luminal ΔpH and NPQ. In this case, it is anticipated that Y(II), Y(NPQ) and Y(NO) will follow the NPQ gradient. (B)–(D) ANOVA statistical analysis was applied to Y(II), Y(NPQ) and Y(NO), respectively, derived from NPQ inductions at 480 s, when the system reached steady state in the T89 genotype. Significant differences are indicated by *, ** and ***, with *p*‐values lower than 0.05, 0.01 and 0.001, respectively. Fluorescence was detected by an arrangement of three fast photodiodes peaking at 686, 700 and 730 nm (10 nm FWHM). (E)–(G) Multidimensional reduction of the data shows differences in Y(II), Y(NPQ) and Y(NO) measured at 686 nm and compared to 700 and 730 nm for each biological replicate. Clustering analysis was performed using the *k*‐means method, with the number of clusters per condition determined by a supervised analysis using the elbow method. Each point represents a distinct biological replicate (*n* > 5). (H) Principal component analysis (PCA) was performed on the differences of Y(II), Y(NO) and Y(NPQ) recorded at 686 nm to 700 and 730 nm. Six variables were computed for each biological replicate. PC1 accounts for 66% of the variation, which is primarily influenced by the differences in Y(NPQ) and Y(NO), as described in the loading plot (I). PC2 explains 30.9% of the variation, mainly driven by differences in Y(II). In (H), each ellipse represents a 95% confidence interval for each genotype based on its centroid. [Color figure can be viewed at wileyonlinelibrary.com]

Figure S13 displays traces of Y(II), Y(NPQ), and Y(NO) calculated from fluorescence inductions detected at three different wavelengths using fast photodiodes (Nanda et al. 2024). As these variables showed a complex behaviour, we next focused our analysis on the final point of the light‐phase induction (Figure 3). We hypothesized that the light gradient would generate a proportional ΔpH gradient, which in turn would translate into a corresponding NPQ gradient (Karabourniotis et al. 2021). Consequently, we expected higher Y(NPQ) values in the upper layers and lower values in the deeper layers. In contrast, based on the general model of energy partitioning, Y(II) should exhibit the opposite trend: fewer excitons are utilized in the upper layers by PSII and more in the deeper layers, reflecting the decrease in NPQ with depth. Additionally, since the fluorescence yield at different detected wavelengths is influenced by re‐absorption, we anticipate that signals measured at 686 nm primarily reflect the upper layers, whereas those at 730 nm are influenced by deeper layers, as is conventionally assumed for PAM fluorometry (Pfündel 2021).

To investigate this, we performed an ANOVA on Y(II), Y(NPQ), and Y(NO) at different detection wavelengths for T89, *psbs*, and *vde* (Figure 3B–D). Y(II) was similar across wavelengths in T89 and *psbs* but decreased at shorter wavelengths in *vde*. In contrast, Y(NPQ) increased at shorter detection wavelengths for T89 and *psbs*, whereas the opposite effect was observed for Y(NO). In *vde*, no significant differences were found for Y(NPQ) and Y(NO) across the detected wavelengths. However, this approach may underestimate the spectral differences between biological samples, as genotype‐specific variations were masked by overall group differences, and a complete analysis of this dataset for a single time point required 63 comparisons (Figures S14 and S15). Accordingly, we designed a novel pipeline applying dimensionality reduction.

Dimensionality reduction is a common technique in data analysis that simplifies complex datasets by reducing the number of variables while preserving essential information (Van Der Maaten et al. 2009). This approach is particularly valuable when dealing with high‐dimensional data, such as that generated by simultaneous detection of fluorescence, where each sample exhibits a heterogeneous spectral signature for Y(II), Y(NO) and Y(NPQ), as defined by Equations ((14), (15), (16), (17), (18), (19)). From these differences, we performed cluster analysis comparing Y(II), Y(NO), and Y(NPQ) at 686 nm with 700 and 730 nm at the end of the NPQ induction (Figure 3E–G). When the cluster analysis was combined with principal component analysis (PCA) to examine the sources of heterogeneity in PSII energy partitioning (Figure 3H,I), 66% of the variation in energy partitioning heterogeneity (PC1) was explained by the antagonistic relationship between Y(NPQ) and Y(NO), while 31% (PC2) was explained by Y(II).

T89, *cao1.3*, and *cao1.11* clustered together in the PCA and cluster analysis, suggesting that a moderate reduction in chlorophyll and LHCII content does not significantly affect how plants cope with increasing light intensities. As expected, these lines were primarily affected in Y(NPQ), likely due to the leaf light gradient. Here, Y(NPQ) showed an antagonist relationship with Y(NO); at 686 nm, larger values of Y(NPQ) were associated with lower Y(NO) values, whereas at longer wavelengths, the opposite trend was observed (Figure 3F,G). In *psbs*, Y(NPQ) was up to 23% larger at 686 nm than 730 nm (Figure S15), exhibiting the most pronounced effect in Y(NPQ). This was accompanied by its antagonistic relationship with Y(NO), like T89 response, a pattern supported by the PCA where their ellipses slightly overlap (Figure 3H). In *vde*, the strongest effect was observed in Y(II), while the typically antagonistic interaction between Y(NO) and Y(NPQ) appeared attenuated. Likewise, the absence of LHCII and the strong reduction in chlorophyll content in *cao2* had only a significant effect on Y(II) heterogeneity (Figure 3I). The cluster analysis indicated that *cao2* exhibited the least heterogeneity in Y(NPQ) and Y(NO), similarly to the effect observed in *vde*, suggesting that some properties might be shared between both lines.

### 3.4 Sustained Quenching Does Not Always Mean Photoinhibition

**Figure 4:**
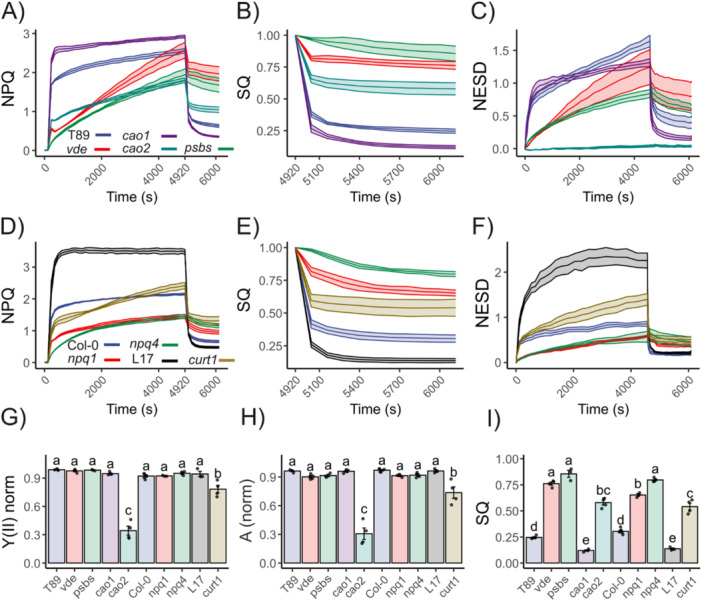
Relationship between NESD, photoinhibition and sustained quenching in aspen and *Arabidopsis* mutants. (A)–(D) Aspens and *Arabidopsis* NPQ inductions by integrated fluorometry (Li‐COR) when illuminated with 1500 μmol photons m^−2^ s^−1^ for 4680 s followed by a relaxation phase of at least 1200 s. Before the light phase, a 240‐s dark interval was recorded to stabilize the sample and determine Fm. (B)–(E) Sustained quenching (SQ) was determined as 1 − (NPQ_max_ − NPQ_t_)/NPQ_max_ from (A) and (D), respectively. (C)–(F) NES development was obtained from spectrally time‐resolved kinetics in similar conditions. (G)–(I) Gas exchange and fluorescence was monitored at 400 ppm and 1500 μmol photons m^−2^ s^−1^ for 4680 s. Each biological replica was normalized to the maximal value of Y(II) and CO_2_ assimilation after full activation of NADPH reductase (≥ 480 s). (G) Normalized Y(II) and (H) CO_2_ assimilation at actinic light end point (4920 s). (I) SQ after 20 min of dark relaxation (6120 s). Shared letters indicate non‐significant differences between the groups (Tukey's tests, *p* < 0.05). Each point represents a distinct biological replicate (*n* > 3). [Color figure can be viewed at wileyonlinelibrary.com]

PCA revealed differences in energy partitioning among the *npq* mutants, with *vde* and *cao2* showing the largest divergence from T89 (Figure 3). Both lines showed a weak antagonist effect between Y(NPQ) and Y(NO) heterogeneity, suggesting that they might be more susceptible to photoinhibition, as is expected from mutants lacking qZ or LHCB proteins (Nilkens et al. 2010; Havaux and Tardy 1997). To investigate the relationships among PSII energy partitioning heterogeneity, NESD, photoinhibition and sustained quenching, we performed a long NPQ induction experiment in HL/photoinhibitory conditions (80 min in 1500 μmol photons m^−2^ s^−1^, 400 ppm CO_2_, 25°C and RH 60%), followed by relaxation for 20 min in our aspen mutants (Figure 4). These experiments combine measurements of CO_2_ assimilation and PAM fluorometry (Li‐6800; Li‐COR). To quantify the amount of quenching that was sustained or slowly relaxing, we used the equation SQ = 1 − ((NPQ_max_ − NPQ_t_)/NPQ_max_), which equates to NPQ normalized. In parallel, we monitored NESD by spectrally resolved fluorescence inductions at 720 nm. Although gas exchange measurements typically are performed using a combination of blue and red light, here we used only red‐actinic light to allow for comparisons and avoid changes in self‐reabsorption during the kinetics.

In this HL regime, we tracked the temporal dynamics of gas exchange. Because photoinhibition produces a de novo quenching mechanism at PSII core (qI; Nawrocki et al. 2021), its progression is expected to reduce PSII quantum efficiency, decrease oxygen evolution rate, and consequently, the activity of the dark reactions. Therefore, once the dark reactions are fully activated, hereafter ~500 s of high light exposure, a time‐resolved decline in CO₂ assimilation (A) under constant light intensity can be interpreted as a proxy of overall photoinhibition, denoted as A_(norm)_ = A_(t)_/A_max_. This concept aligns with the approach described by Adams et al. (2013), in which changes in CO_2_ uptake are tracked across treatments to evaluate photoinhibition. During these experiments, T89, *psbs* and *vde* kept up CO_2_ assimilation throughout the experiment (*psbs* and *vde* somewhat lower than T89 and *cao1.11*, hereafter *cao1*, higher than T89), showing only a 4% to 8% decline in A_(norm)_, whereas in *cao2.18* (hereafter *cao2*) it decreased by 70% (Figures 4H and S18). During this treatment, also, *psbs* and *vde* developed significant quenching (Figure 4A); at the end of the induction phase, *vde* had even higher NPQ than T89. This quenching relaxed slowly, NPQ decreased only by 20%–25% in *psbs* and *vde* compared to 75% in T89, while *cao2* showed a 40% decrease (Figure 4B,I). Under these conditions, *psbs* and *vde* also developed NES (Figures 4C and S16), albeit significantly less than T89, whereas in *cao2* the NES was absent. Hence, *cao2* suffered from photoinhibition during these conditions without developing NES, while *psbs* and *vde* developed sustained quenching and NES with photoinhibition comparable to T89 (Figure 4H). Although no changes were observed in stomatal conductance (gsw) for all the lines analyzed (Figure S18), the intracellular CO_2_ (Ci) increased up to 25% in *cao2*, suggesting an independent regulation of stomatal closure by CO_2_ signalling in *cao2* (Engineer et al. 2016). Under these conditions, *cao1* exceeded the NPQ capacity and its relaxation when compared with T89.

To confirm the unexpected results of *psbs* and *vde* in another angiosperm species, we conducted a long‐term NPQ kinetics (same conditions as described above) experiment using *Arabidopsis* lacking PsbS (*npq4*) or VDE (*npq1*), or overexpressing PsbS (L17). To see if thylakoid structure was important for these traits, we also included the quadruple mutant of CURVATURE THYLAKOID 1, *curt1*, strongly affected in thylakoid stacking and plasticity. The results were similar, CO_2_ assimilation was kept up, with a 3%–8% decline in A_(norm)_, in Col‐0, *npq4* and *npq1* after extended exposure to HL, and like in aspen, the quenching was only slowly relaxing (Figures 4D,E,H and S16). NES were developed in all genotypes, where L17 reached values up to 2 and large sustained values in the relaxation phase (Figures 4F, S17 and S19). Finally, *curt1* had a strong NESD and significant sustained quenching, but up to 50% decrease in CO_2_ assimilation and Y(II) (Figure 4G,H). Under these conditions, no significant decrease – but a tendency – was observed in gsw for L17, whereas *curt1* showed a 50% decline compared to Col‐0, indicating that the decline in CO_2_ assimilation in *curt1* could be linked to the redox state (Wang et al. 2016). Accordingly, *curt1* showed a decline of 30% in qP when compared to Col‐0 at the end of the treatment (Figure S16). Lastly, *cao2* exhibited a 150% increase in Y(NO) during the relaxation phase compared to its reference value in dark‐adapted state, while *curt1* showed similar values (Figures S18 and S19).

**Figure 5:**
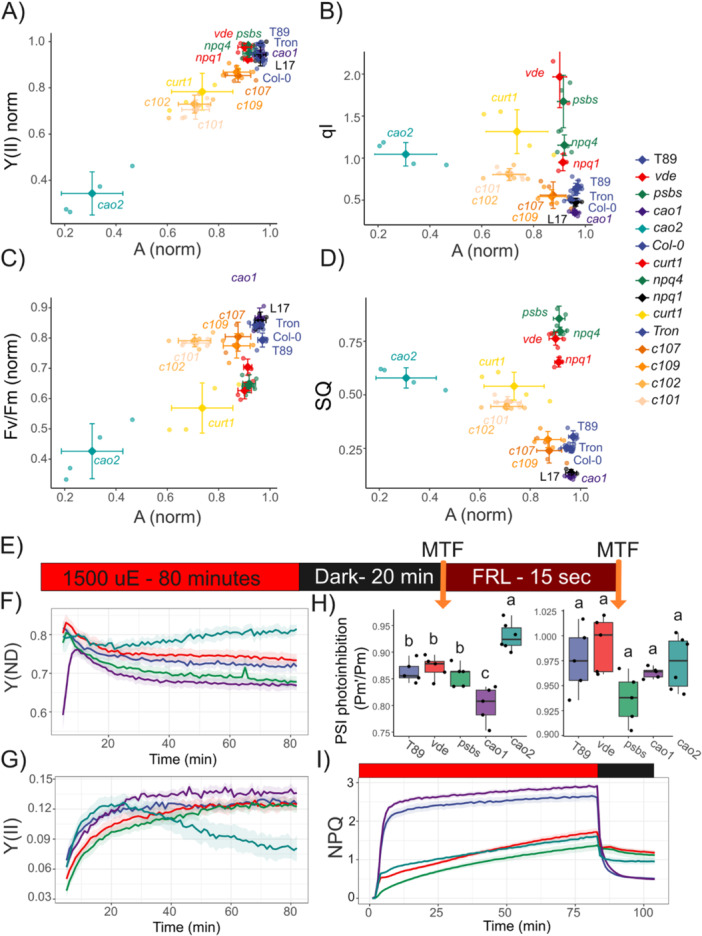
Sustained quenching does not always mean photoinhibition. (A)–(D) Aspens, *Arabidopsis* and barley chlorophyll a oxygenase mutants NPQ inductions by integrated fluorometry when illuminated with 1500 μmol photons m^−2^ s^−1^ for 4680 s followed by a relaxation phase of 1200 s at 21% O_2_ and 400 ppm of CO_2_. (A) Relation between normalized Y(II) and CO_2_ assimilation at actinic light end point (4920 s) to their respective maximal values obtained during the high light treatment. (B) qI was calculated as (Fm − Fm″)/Fm″ at the end point of the relaxation phase (6920 s) and compared to normalized A. (C) Fv/Fm was calculated at the end point of the relaxation phase and normalized to Fv/Fm recorded in the dark‐adapted state. (D) Sustained quenching (SQ) was determined as 1 − ((NPQ_max_ − NPQ_t_)/NPQ_max_) at the end of the relaxation phase and compared to normalized A. (E) Diagram of Aspens NPQ inductions by integrated fluorometry and P700 pulse method (Dual‐PAM‐100) performed at 1500 μmol photons m^−2^ s^−1^ for 4800 s followed by a relaxation phase of 1200 s in dark. At the end of the kinetic, a far‐red light pulse (250 μmol photons m^−2^ s^−1^) of 15 s was applied to fully oxidized P700. (F) and (G) Y(ND) and Y(II) kinetics for aspens npq mutants during the high light treatment. Note the antagonistic relationship between Y(II) and Y(ND) in *cao2*. (H) PSI photoinhibition was measured after 20 min of dark relaxation by the P700 pulse method (MTF) and after a FRL pulse followed by an MTF. (I) NPQ kinetics. Data are mean ± SD (*n* = 5 biologically independent experiments). [Color figure can be viewed at wileyonlinelibrary.com]

To highlight the marked differences between SQ, Y(II), and A (Figure 5), we evaluated how well classical parameters for estimating photoinhibition reflect changes in CO_2_ assimilation. Moreover, we performed the same photoinhibtory treatments in a collection of *cao* mutants from barley (Bossmann et al. 1997; Mueller et al. 2012) to evaluate the relation between smaller PSII antenna size and photoinhibition (Figure S20). Here, a pronounced reduction of 50% to 100% of Lhcb1‐3 enhanced susceptibility to HL treatments in *cao* mutants when compared to Tron (barley reference line, Figure S21). Interestingly, *cao2* counterparts in barley (*c102‐c101*) showed a decline in A_(norm)_ of ~30%–40%, suggesting a species‐dependent susceptibility to photoinhibition for *cao* mutants, whereas c*107‐c109*, with a pronounced reduction in Lhcb and more like *cao1*, showed only a ~10%–15% decline in A_(norm)_. Overall, we found that Y(II)_norm_ correlated strongly with A_(norm)_, with an *R*² of 0.92 (*p* = 2.2 × 10^−16^). In contrast, no correlation was observed between A_(norm)_ and either qI (Equation 20) or SQ. Fv/Fm, commonly employed to monitor PSII damage (Nawrocki et al. 2021), showed a moderate correlation with A_(norm)_ decline (*R*² = 0.47, *p* = 1.29 × 10^−9^).

Additionally, we assessed the effect of photoinhibition on PSI during HL treatments in aspen, barley and *Arabidopsis* mutants (Figures 5, S22 and S23). To do this, Pm was determined by applying a strong FRL pulse to dark‐adapted leaves, followed by a SP. After photoinhibitory treatment, Pm′ was measured both with and without the FRL pulse. We found that, without proper oxidation of PSI, PSI photoinhibition can be overestimated by up to 20% under HL conditions in aspens, barley and *Arabidopsis*. In contrast, full oxidation of the system, necessary to properly achieve Pm′, indicates the absence of PSI photoinhibition during these treatments in T89 and Tron. In *Arabidopsis*, a ~5%–8% of PSI photoinhibition was observed in Col‐0 and its respective qE mutants. During these kinetics, we monitored Y(ND) and Y(II), which revealed that photoinhibition in *cao2, c101 and c102* led to a decline in Y(II), accompanied by a progressive increase in Y(ND) over time. In contrast, qE mutants in *Arabidopsis* and aspens showed a progressive increase in Y(II), followed by a reduction of Y(ND).

**Figure 6:**
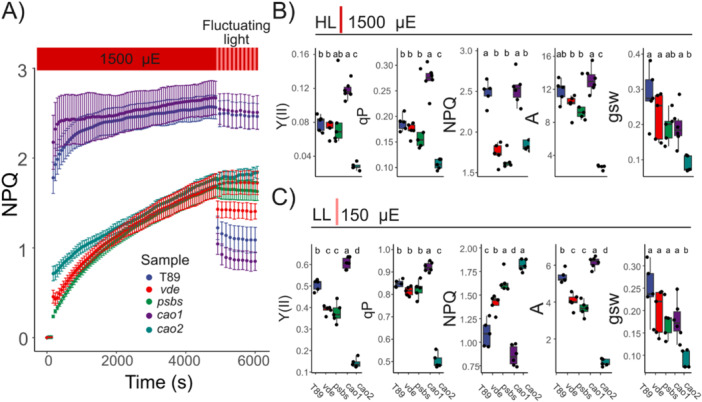
Sustained quenching compromises A and Y(II) under LL in dynamic conditions. (A) NPQ inductions under illumination at 1500 μmol photons m^−2^ s^−1^ for 4680 s, followed by a fluctuating phase alternating between 150 and 1500 μmol photons m^−2^ s^−1^ with 60 s step duration. Low‐light (LL) periods (150 μmol photons m^−2^ s^−1^) are indicated in light red (top), while high‐light (HL) periods (1500 μmol photons m^−2^ s^−1^) are shown in red. Gas exchange and fluorescence were monitored at 21% O_2_ and 400 ppm of CO_2_. Values of Y(II), qP, NPQ, A, and gsw from the last five steps were integrated for each biological replicate in LL (B) or HL (C). Shared letters indicate non‐significant differences between groups (Tukey's test, *p* < 0.05). Each point represents a distinct biological replicate (*n* > 5). T89, *vde* and *psbs* are coloured in blue, red and green, respectively. [Color figure can be viewed at wileyonlinelibrary.com]

Lastly, we hypothesized that the sustained quenching phenotype would impair performance under dynamic conditions; therefore, we investigated its impact during a fluctuating light protocol (150–1500 μmol photons m^−2^ s^−1^ steps; 20 min × 1 min step) in aspen *npq* and *cao* mutants (Figure 6). In dynamic responses following sustained quenching, T89 exhibited 25%–33% higher A and 22%–25% higher Y(II) during the low‐light steps compared with *vde* and *psbs*, respectively. During the HL steps A, qP and Y(II) remain unchanged. NPQ had the largest effect during the low‐light (LL) steps, where *psbs* and *vde* showed 48% and 28% increases in NPQ compared to T89. Surprisingly, *cao1* was able to outperform T89, showing an increase of ~50% in Y(II) during the HL phase and ~12% in A and ~20% in Y(II) during the LL steps.

**Figure 7:**
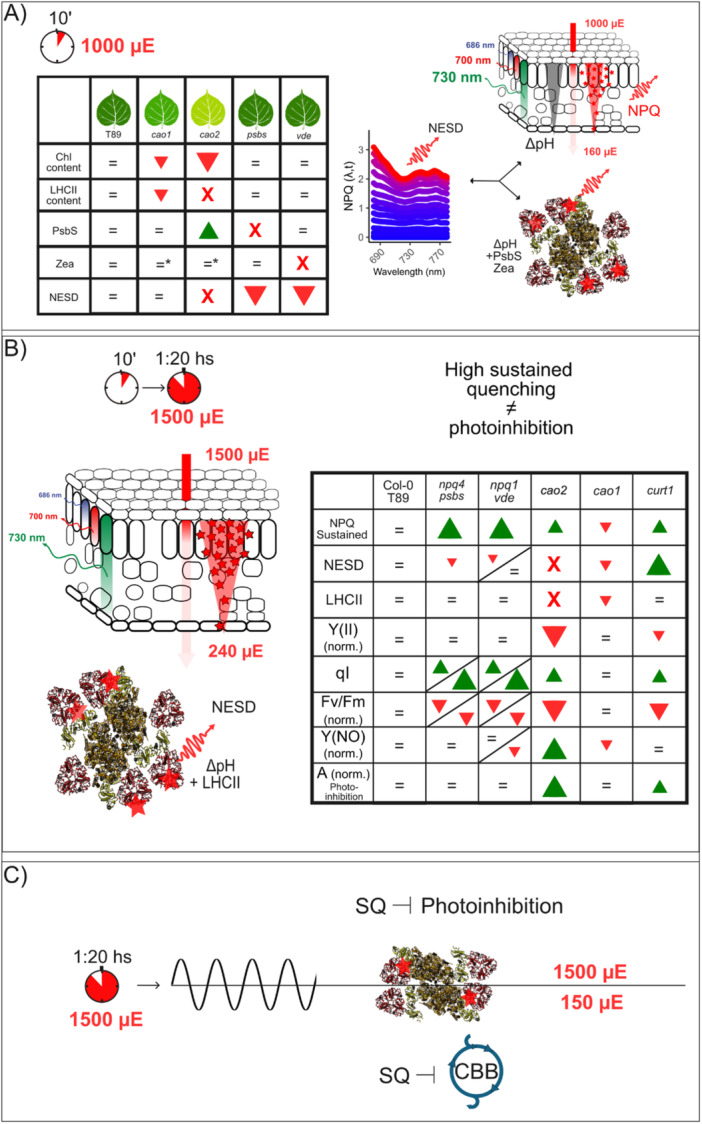
The relations between NESD, photoinhibition and sustained quenching. (A) Table summarizing the relationships between chlorophyll content (Chl), LHCII content, PsbS, zeaxanthin formation (Zea), and the development of new emitting species (NESD) in response to fast NPQ inductions within the leaf. The red triangles and their respective sizes indicate the direction and magnitude (decrease) of phenotypic changes relative to the T89 genotype. Red crosses denote the absence of the specified variable. A schematic illustrating the relationships between NPQ gradient (represented by a red triangle), light gradient (depicted by a red arrow), and the quenching activities of Zea, PsbS and LHCII at photosystem II (red stars). The interplay of these factors leads to a novel property of NPQ characterized by a significant reduction of NPQ above 700 nm, associated with the emergence of NES. In Zea, asterisks denote unchanged VDE activity resulting from qE inductions. (B) Under sustained quenching, the development of NESD is independent of PsbS and Zea, where the only pre‐requisite is the presence of LHCII proteins. A table summarizing the relationships between sustained quenching (NPQ sustained), NESD, LHCII, Y(II), qI, Fv/Fm, Y(NO) and photoinhibition is presented. Here, Y(II) norm. shows changes at the end of the actinic light period compared to maximal Y(II) obtained during the time‐resolved kinetic. Y(NO) norm. and Fv/Fm norm. represent values obtained at the end of the relaxation phase and normalized to the dark‐adapted values. Note that Y(II), being derived from the light‐adapted state, serves as a good proxy of photoinhibition when compared to qI or Fv/Fm, which are co‐dependent on Fm. Y(NO) reflects changes in Fo during the relaxation phase. The red triangles indicate a decrease compared to their reference line, whereas the green triangles indicate an increase. (C) Under dynamic conditions, sustained quenching has a protective role under increasing light intensities at the expense of excessive photoprotection during low light intensity periods. [Color figure can be viewed at wileyonlinelibrary.com]

The differences in these traits across mutants lacking different proteins are summarized under standard conditions (Figure 7A), under photoinhibitory conditions (Figure 7B), and under dynamic conditions (Figure 7C).

## 4 Discussion

### 4.1 What Produces NES During NPQ?

In this work, we introduce the first collection of *cao* mutants produced in a tree, where CAO activity was partially (*cao1*) or completely disrupted (*cao2*) in aspen, decreasing chlorophyll b content and LHCII complexes stability (Figure 1). Although it is generally believed that LHCII abundance affects thylakoid stacking (Kim et al. 2009), their absence does not affect stacking in aspens and *Arabidopsis* (Figure 1; Nicol et al. 2019). It has been proposed that Lhcb5 in the absence of LHCII could play a compensatory role for thylakoid stacking, even in the absence of chlorophyll b (Guardini et al. 2022; Guardini et al. 2025). Similarly, barley *chlorina*‐*f2* shows larger stacking than a reference line, suggesting a much more complex relationship between LHCII and grana stacking (Bassi et al. 1985). In aspen, we observed that when chlorophyll b synthesis was disrupted, not only Lhcb5 but also Lhcb4 is stable, like in barley *chlorina*‐*f2* (Bossmann et al. 1997). Obviously, the stability of these proteins in the absence of chlorophyll b differs between species, and *cao* mutations do not necessarily lead to LHC‐less plants (Guardini et al. 2025). Lhcb4 and Lhcb5 might have a role in thylakoid stacking when LHCII is absent, producing the unaltered stacking phenotype observed in *cao2*.

The apparent increase in PSII and PSI RC in *cao2* mutants is a consequence of the normalization of gel loading on an equal chlorophyll basis (Figure 1), a pronounced decrease of LHCII in *cao2* leads to a relative increase in the abundance of core proteins. In *cao1*, however, PSI is more abundant, indicating a potential imbalance between PSII and PSI compared to T89. Similar effects have been reported in barley and *Arabidopsis cao* mutants (Andrews et al. 1995; Guardini et al. 2025). Moreover, although NPQ is largely dependent on LHCII complexes, the fast induction of the qE component present in aspens *cao2*, as in *ch1*, suggests that PsbS and Zea can function in the absence of LHCII, potentially targeting the remaining functional minor antenna (Guardini et al. 2025). Another notable feature of *cao2* is the larger amount of ATP synthase. In *cao*, the smaller investment of N in LHC complexes could result in more N available for enhancing other enzyme activities by increasing protein abundance (Song et al. 2017). However, it remains unclear whether the pronounced increase in ATPC abundance results in enhanced ATP synthase activity and, consequently, in a reduction of the proton motive force across the thylakoid membrane, which could influence the NPQ phenotype of *cao2* (Ermakova et al. 2022). Importantly, ATP synthase abundance does not necessarily imply a corresponding increase in enzymatic activity, as ATP synthase is subject to post‐translational regulation, and a substantial fraction of the enzyme may remain inactive under our studied conditions (Bunney et al. 2001; Rott et al. 2011; Schöttler and Tóth 2014; Strand et al. 2023).

Perhaps one of the most unclear/controversial features of *cao* mutants is their susceptibility to photoinhibition. In algae, it has been shown that a smaller antenna leads to less photoinhibition (Neidhardt et al. 1998; Polle et al. 2003), in plants, the situation may be opposite (Havaux and Tardy 1997; Kim et al. 2009; Mao et al. 2025). Some of these discrepancies may arise from different definitions of photoinhibition, frequently confounded with sustained photoprotective quenching in fluorescence studies (Malnoë 2018). In recent years, the study of photoprotective sustained quenching and photoinhibition has gained a lot of attention (Malnoë et al. 2018; Bag et al. 2020; Nawrocki et al. 2021; Bru et al. 2022; Smith et al. 2024). Although these processes are important for ecosystems and agricultural productivity, they are quite poorly understood, and their definitions from time‐course fluorescence data after certain treatments are not straightforward. Malnoë (2018) argues that qI has rightfully been called ‘ill‐defined’ (Nilkens et al. 2010) and the source of confusion in the field comes from the term ‘photoinhibition’ as Maxwell and Johnson (2000) explained: ‘it is important to note that this term, when applied to fluorescence analysis, generally refers to both protective processes and to damage to the RCs of PSII (Osmond 1994), while in more molecular studies, it is specifically the latter that is referred to as photoinhibition’. If we regard qI‐photoinhibition as quenching at the core of PSII (Nawrocki et al. 2021), qI occurs when the photoprotective machinery has failed to protect PSII and represents a costly mechanism, negatively affecting oxygen evolution and productivity. Sustained photoprotective quenching is one of the mechanisms that protects PSII RC from photodamage and, therefore, could prevent the development of qI. In plants, the antenna plays a pivotal role in balancing light harvesting and quenching, and it can be assumed that NPQ, including sustained photoprotective quenching, involves LHCB complexes (Ruban and Saccon 2022; Bru et al. 2022). We have also reported one sustained photoprotective quenching mechanism in overwintering gymnosperms, PSII protection by direct energy transfer to PSI (Bag et al. 2020). Although it is possible that such a mechanism may also be present in angiosperms (see Nanda et al. 2025 for discussion and references), there is likely a mechanistic diversity of sustained photoprotective quenching across plant lineages.

By using spectrally resolved fluorescence and net photochemical productivity, monitored by gas exchange analysis, we demonstrate when photoinhibition is coupled or not to sustained photoprotective quenching, and the spectral signatures associated with these processes. Under increasing light and in the absence of photoinhibition, plants develop so‐called NES (Figure 2), and two models have tried to explain their origin. The idea that NES are linked to qE/PsbS/LHCII (see, e.g., Horton et al. 2005; Holzwarth et al. 2009; Johnson and Ruban 2009) has been challenged by recent studies, suggesting that their origin is a product of closed PSII RC under actinic light (Farooq et al. 2018). Here, we explored in the absence of photoinhibition how the decrease of PSII absorption cross‐section and the absence of NPQ modulators affect the development of NPQ spectra, focusing on aspens. Upon NPQ induction, T89 shows a classical NPQ evolution spectrum, in which NES are observed at longer wavelengths (Figure 2). We also show that when comparing reference lines and *npq* mutants, differences in the emission spectra can lead to an underestimation of NPQ by up to 50% when using integrated methods (Figures S10 and S11).

We suggest a new parameter, NESD, that can be calculated from this type of data to obtain time resolution of spectral differences in NPQ. This parameter captures the dynamics of the process, an aspect that cannot be monitored using ultrafast time‐resolved spectroscopy, which measures chlorophyll fluorescence lifetimes under steady‐state conditions (Chukhutsina et al. 2019). In native systems, NES are rapidly both induced and relaxed, exhibiting kinetics similar to those of qE development (Figures 2 and S9). Such fast relaxation would not make comparable the PSII emission spectra in the closed state during NPQ and the open state during the relaxation phase, like in Farooq et al. (2018). Moreover, we found no NESD in c*ao2* mutants lacking LHCII; if closed PSII RCs were the source of NES, then *cao2* would be expected to show heterogeneous NPQ spectral profiles over time. Instead, their spectra remain uniform (Figures 2 and 4). Taken together, these observations support the conclusion that, under NPQ conditions, it is unlikely that closed PSII RCs are the source of NES. Under long NPQ inductions at 1500 μE, where significant sustained quenching was established in T89, NESD could only be detected after 2 min of dark relaxation, was nearly absent in the Col‐0 line but enhanced in L17 overexpressing PsbS (Figures 4 and S24). This suggests that the findings from Farooq et al. (2018) might be due to more complex emerging properties.

### 4.2 Light Gradient, qE and PSII Energy Partitioning Heterogeneity – A Complex Relationship

NPQ spectrum and NESD in the leaf seem to be connected to PsbS, Zea, NPQ and photoprotection, but how? We think we could rule out the possibility that the NPQ spectrum and NES are affected by self‐absorption in the leaf, based on the arguments around Equation (12) above. However, NES might develop as a consequence of differential excitation within the leaf, known as the light gradient (Figure 3A). When light strikes the upper surface of a leaf, it is not distributed evenly across all tissue layers (Karabourniotis et al. 2020). Photosystems located in the upper layers of the leaf absorb more light and are more excited than those at deeper layers. Thus, the light gradient produces an NPQ gradient across the leaf. As a result, NESD may manifest as a gradual decline in qE with depth, which cannot be corrected using Equation (3).

However, if the light gradient were the sole explanation for NESD, *psbs*, *vde* and T89 – having similar chlorophyll content – would be expected to exhibit comparable NESD. We observed that *vde* can exhibit negative NESD when comparing NPQ at 686 and 740 nm, indicating that attributing NESD solely to the light gradient is not sufficient (Figure 2). T89 and *cao1* display comparable NESD, although *cao1* has a reduction in chlorophyll content, which would typically produce a more uniform light gradient. Instead, our data suggests that NPQ and NESD should be considered emergent properties resulting from the interplay between the light gradient and changes in thylakoid reorganization, which together influence chlorophyll fluorescence yields during NPQ and its apparent spectrum. These emergent properties ultimately influence the fate of absorbed excitation energy across wavelengths, manifesting as heterogeneity in PSII energy partitioning (Figure 3).

Under our experimental conditions, greater discrepancies in PSII energy partitioning heterogeneity were observed among T89, *psbs*, *cao2*, and *vde*. The light gradient appears to create an antagonistic relationship between Y(NPQ) and Y(NO), while Y(II) remains relatively unaffected in T89 (Figure 3). This suggests that Y(II) is evenly distributed across different chloroplast layers, maintaining consistent quantum efficiency performance. Additionally, the higher Y(NPQ) and lower Y(NO) values observed at 686 nm indicate an appropriate response to the increased excitonic pressure at the upper surface of the leaf. As expected, a similar response was seen in *cao1*, and surprisingly, also in *psbs*, indicating that these mutants effectively respond to the light gradient. However, *vde* and *cao2* exhibited a different pattern, where primarily Y(II) was influenced, and the antagonistic relationship between Y(NPQ) and Y(NO) heterogeneity was largely reduced, although *vde* had a chlorophyll content like T89 (Figure 1). Overall, these findings highlight the importance of simultaneous chlorophyll fluorescence detection to gain a deeper understanding of PSII energy partitioning heterogeneity in plants, a process influenced by the interaction between the light gradient and NPQ subprocesses.

These findings underscore an important technical consideration: fluorescence signals at shorter wavelengths are generally excluded because they are biased toward processes in the upper leaf layers (Pfündel 2021). Spectral resolution, however, retains this information while preserving contributions from longer wavelengths. In other words, spectrally time‐resolved chlorophyll fluorescence enables a spectral deconvolution of a scenario that conventional chlorophyll fluorometry oversimplifies. Thus, when NPQ is analyzed at shorter wavelengths, it becomes evident that traditional methods substantially underestimate NPQ capacity, since upper‐layer information is preserved and PSII chlorophyll fluorescence quenching remains largely unaffected by NES development (Figure 2).

What, however, needs to be clarified is whether these complex spectral signatures could also result from heterogeneity in chloroplast response, influenced by the light gradient. This would align with discrepancies observed in the NPQ–photochemistry relationship of diatoms, potentially stemming from overlooked assumptions such as cellular heterogeneity (Croteau et al. 2025).

### 4.3 When Sustained NPQ Uncouples From Photoinhibition

Lack of qE will increase excitonic pressure on PSII and influence the RC state, increasing the formation of a slow NPQ component named δ by Ramakers et al. (2025), assumed to represent qI or a qI‐like process (Li et al. 2002; Nilkens et al. 2010). qI would affect the rate of oxygen evolution and therefore CO_2_ assimilation (Nawrocki et al. 2021). We observed, however, in the absence of PsbS or VDE, hence qE, in both aspen and *Arabidopsis*, significant sustained quenching but only a small effect on CO_2_ assimilation (Figure 5). This is inconsistent with the idea that the slowly arising component in *npq1* during extended NPQ induction is strictly connected to qI (Nilkens et al. 2010). Despite early studies reporting a strong photoinhibition in *npq4* and *npq1* (Havaux and Niyogi 1999; Li et al. 2002), under our experimental conditions with only red‐actinic light for 80 min we observe a small decrease in CO₂ assimilation during the rise of the sustained quenching, hence the increase in sustained quenching is unlikely to be solely due to increasing qI. In contrast, *cao2* showed a 75% decrease in CO_2_ assimilation as well as Y(II) decline, and a large amount of sustained quenching without the production of NES, supporting the suggestion of Nawrocki et al. (2021) that qI takes place at PSII RCs without any spectral signatures. Similarly, in barley *cao* mutants, the progressive decline of PSII antenna enhanced susceptibility to HL, although *cao1* showed better performance than the reference line T89. This suggests that optimal tuning of LHCII content can be achieved by manipulating chlorophyll b content without the classic associated drawbacks of *cao* mutations (Havaux and Tardy 1997; Kim et al. 2009).

Moreover, by using *curt1*, impaired in thylakoid plasticity, we found that thylakoid reorganization is important for a proper response to increasing light (Garty et al. 2024; Nanda et al. 2025). In *curt1*, this impairment was associated with a marked decline in CO₂ assimilation and Y(II), elevated levels of NESD, and a slow induction of sustained NPQ (Figure 4H–K). Interestingly, stomatal conductance was affected in *curt1*, likely associated with an over‐reduced PQ pool assessed from qP (Figure S19), a proposed cue for stomatal closure (Wang et al. 2016). Accordingly, *curt1* is characterized by an inefficient electron transport process, whereas the larger the grana diameter, the more reduced is the PQ pool due to constrained diffusion of electron carriers between photosystems (Armbruster et al. 2013; Höhner et al. 2020).

We contrasted classic fluorescence parameters, such as qI or Fv/Fm, to the decline of assimilation during the kinetic (Figure 5). Both parameters largely overestimated the extent of photoinhibition in qE mutants, where photoprotective sustained quenching was developed. Although Y(NO) remains a poorly understood parameter, it can be used to monitor the relation between the minimal yield of fluorescence at any given time and its maximum in the dark‐adapted state (Equation 7). Thus, any increase in Y(NO) between the dark‐relaxation phase and the dark‐adapted state reflects changes at the minimal yield of fluorescence product of impaired photochemistry or antenna detachment. Since *cao2* does not exhibit the latter, the rise in Y(NO) points to the accumulation of inactive, damaged PSII RC, consistent with the overall 70% reduction in A_(norm)_ (Figure 4).

We found that Y(II) decline is a good proxy of photoinhibition during long NPQ inductions, which turns out to be a more accurate estimator than other classic estimators like qI or Fv/Fm, especially in the presence of photoprotective sustained quenching (Figure 5). Interestingly, Y(II) is a parameter derived from the light‐adapted state, whereas qI and Fv/Fm are co‐dependent on Fm. In recent years, extensive discussion about the presence of PSII conformational changes during the transition from the dark‐adapted state to the light‐adapted state has raised concerns regarding the feasibility of the Qa model (Sipka et al. 2021; Garab et al. 2023). Thus, it is possible that some of the above‐mentioned discrepancies surrounding Fv/Fm and qI and their feasibility in discriminating photoinhibition from sustained photoprotective quenching could be linked to the complex nature of chlorophyll a variable fluorescence.

When monitoring Y(II) and PSI simultaneously, a decline in Y(II) was antagonistically associated with Y(ND), particularly when LHCII abundance was strongly affected (Figures 5 and S23). In other words, not meeting PSI electron demand, and thus reinforcing PSI donor‐side limitation, appears to be a ‘safe mode’ that the system adopts once sustained photoprotective quenching is no longer effective. Oppositely, in the absence of qE (*npq1*, *npq4*, *vde*, and *psbs* mutants), Y(II) slowly increased while Y(ND) decreased during the kinetics. This indicates not only a lack of evident photoinhibition during HL but also the presence of a slow adjustment in which PSI electron demand is met by PSII charge separation, even in the absence of qE.

When assessing PSI photoinhibition at the end of the treatment, we initially obtained values that are consistent with previous reports in which weak SP or undefined FRL intensity were applied (Mao et al. 2025). On the contrary, with a strong SP and FRL pulse, PSI photoinhibition was completely absent in aspens and barley, regardless of whether qE or LHCII were present. In addition, *Arabidopsis* showed a similar pattern, with the same extent of PSI photoinhibition independently of qE (Figures S22 and S23). Therefore, we recommend using strong FRL pulses together with strong SP pulses to ensure full oxidation of P700, avoiding PSI photoinhibition overestimations. Throughout evolution, PSI has co‐opted a plethora of alternative electron‐acceptor pathways to prevent over‐reduction of its acceptor side, making PSI photoinhibition unlikely under our experimental conditions, regardless of qE. However, further research is needed to clarify how qE interacts with other stress conditions where PSI photoinhibition has been observed (Takeuchi et al. 2025) and if the susceptibility to PSI photodamage is dependent on species and life history.

Previous studies have reported that Y(II) remains unaffected under increasing light intensities in both *npq1* and *npq4* mutants, even under HL treatments like those applied in this study (Külheim and Jansson 2005; Ware et al. 2015). Conversely, no changes in CO_2_ assimilation were observed in Col‐0 and *npq4* plants subjected to cycles of 100/1000 μE light fluctuations, and no effects were found on biomass production under several fluctuating light conditions (Schiphorst et al. 2023). We observed a large amount of sustained quenching in classical *npq* mutants, evoking the original conclusions about *npq4* from Johnson and Ruban (2011). Thus, PsbS and Zea are most likely essential to rapidly fine‐tune the rate of energy transfer from the antenna to the RC upon NPQ, but their requirement is not mandatory. Under natural conditions, *npq1* and *npq4* mutants can cope with light fluctuations with moderate effects at the fitness level (Külheim et al. 2002), whereas the absence of other regulatory processes involved in photosynthetic control may have more drastic effects. Proton Gradient Regulator 5, involved in the regulation of cyclic electron flow, is, for example, more important (Suorsa et al. 2012). Mutants lacking *curt1* possess the fast photoprotective mechanisms associated with LHCII, but the absence of thylakoid plasticity results in reduced long‐term responses to HL and fluctuating light environments (Pribil et al. 2018). This suggests that an interaction between NPQ and thylakoid rearrangements is necessary for plants to cope with changing light intensities. What remains to be understood is the relevance of thylakoid structural re‐arrangements and to what extent the lateral heterogeneity applies in the light‐adapted state where photosynthesis takes place (Andersson and Anderson 1980; Garty et al. 2024; Nanda et al. 2025).

Our results suggest that slow photoprotective subprocesses in *npq* mutants are also dependent on LHCII and PsbS, and Zea are catalysts of these subprocesses instead of direct quenchers (Figure 6). When qE catalyzers are absent, although a large amount of photoprotective quenching can be achieved, the lack of fine‐tuning has a direct tradeoff in CO_2_ assimilation under dynamic conditions, a feature targeted to enhance photosynthesis in natural conditions (Kromdijk et al. 2016; De Souza et al. 2022). Thus, this slowly relaxing sustained quenching, involving antenna components, can help plants to cope with HL but at the expense of larger penalties in dynamic conditions (Figures 6 and 7C). qH has been suggested to be a form of sustained photoprotective quenching mechanism dependent on LHCII upon long actinic light exposure (Bru et al. 2022). Maybe qE, qZ, and qH and other potential photoprotective mechanisms, including spillover from PSII to PSI (Bag et al. 2020), should be viewed not as separate mechanisms with distinct targets, but as overlapping components of a – yet not understood – holistic regulatory process that collectively modulate the functional antenna size of PSII, primarily by acting at the LHCII complexes.

In conclusion, a deeper understanding of photoinhibition requires integrating multiple complementary methods. Relying solely on steady‐state chlorophyll fluorescence parameters, such as Fv/Fm or qI, can lead to over‐simplified interpretations (Müller et al. 2001; Demmig‐Adams et al. 2006; Malnoë 2018), because many distinct mechanisms influence the lifetime of PSII in its open and closed states and therefore minimal and maximal fluorescence yields. In the absence of ultrafast time‐resolved methods, processes like qE, antenna uncoupling, the presence of damaged PSII RCs, sustained photoprotective quenching, and PSII photoinhibition can be easily conflated. Similarly, exclusive dependence on NPQ kinetics poses comparable limitations. Apparent contradictions, such as the slow‐rising NPQ component in qE mutants versus the rapid and seemingly larger NPQ capacity of *cao* mutants, can lead to misinterpretation, especially when analyses are restricted to conventional 10‐min inductions. Furthermore, it is essential to clearly define how PSI photoinhibition is assessed, since changes during photoinhibition can alter PSI charge‐separation properties and result in overestimations. Therefore, incorporating time‐resolved CO₂ assimilation provides the necessary physiological context for interpreting photoinhibition at the light reactions. Integrating these signals allows a clearer and more accessible distinction between true photoinhibition and other processes, such as photoprotective sustained NPQ.

## Supporting information

Supplementary Cainzos et al.

## Data Availability

The data that support the findings of this study are available from the corresponding author upon reasonable request.
