# Supplementary material for: Loss of qE Does Not Necessarily Lead to Photoinhibition: Sustained Non‐Photochemical Quenching in the Absence of PsbS and Zeaxanthin
Source: Plant Cell Environ. 2026 Mar 8;49(6):3405–25. doi: 10.1111/pce.70477 (PMC13136559; doi:10.1111/pce.70477)
Supplement: Supplementary file 1 — Supplementary Cainzos et al. [file PCE-49-3405-s001.docx]

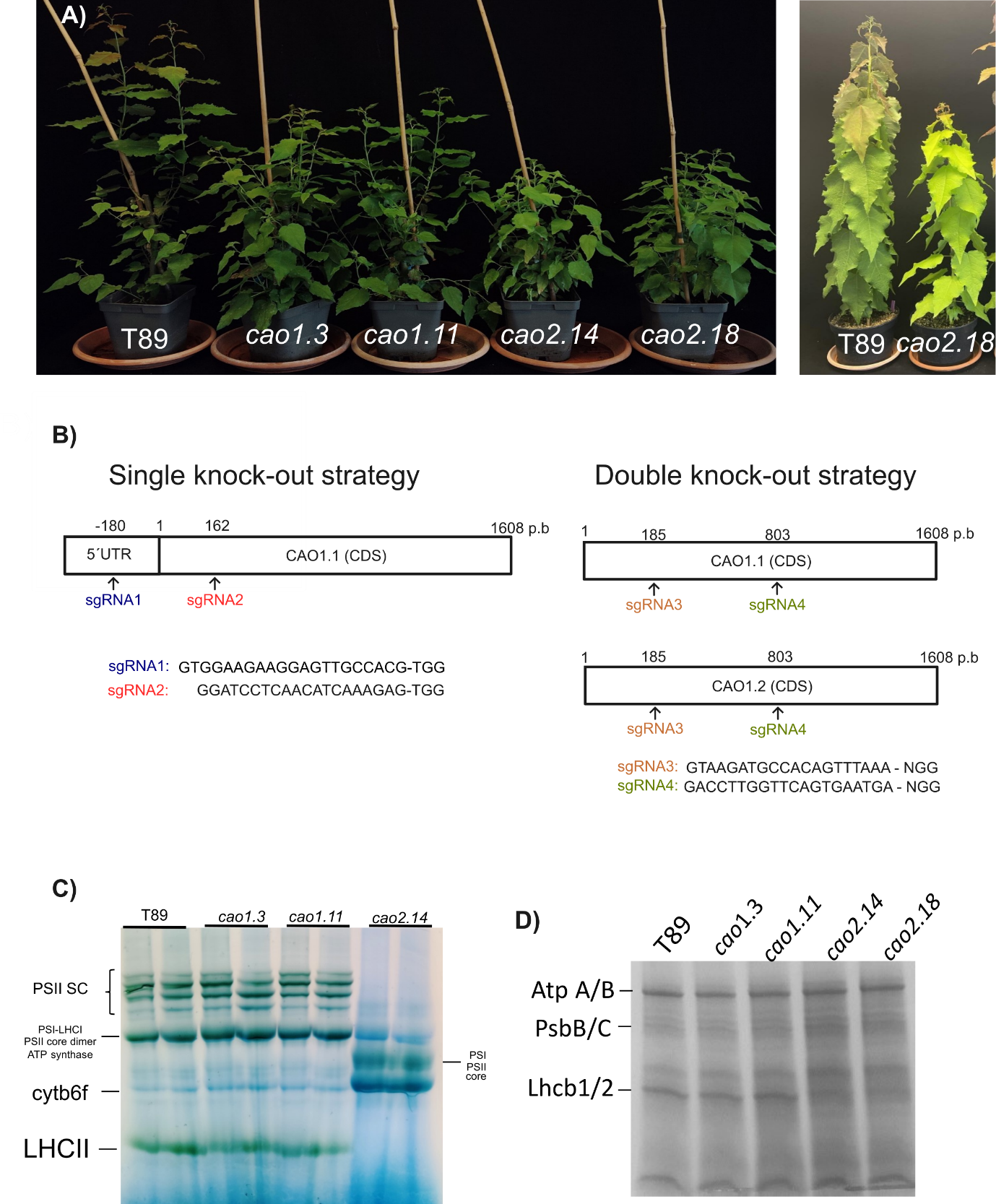


**Supplementary Figure 1. Collection of aspen chlorina mutants.** A) In the left panel, examples of young T89, *cao1.3*, *cao1.11*, *cao2.14* and *cao2.18* are shown. In the right panel, examples of adult T89 and *cao2.14* are shown. C) Molecular cloning design**.** sgRNA’s for CAO1.1 and CAO1.1 + CAO1.2 were designed to knock out one (*cao1*) or both genes (*cao2*). D) BN-PAGE from aspens thylakoids solubilized in 2% b-DM. E) SDS-PAGE from aspens thylakoids. 2 μg of chlorophylls were loaded per lane for each gel.

**
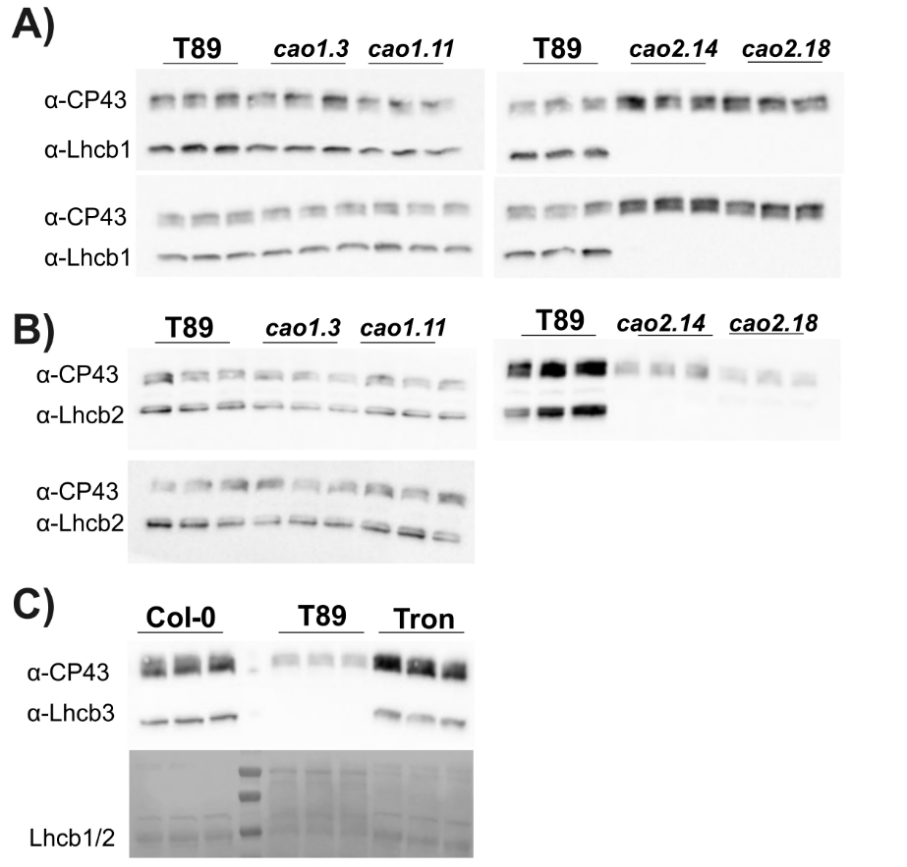
**

**Supplementary Figure 2. Immunoblots of Lhcb proteins in T89 and *cao* mutants.** A) 2 μg of chlorophylls were loaded into SDS-Page and blotted against α-CP43 and α-Lhcb1 for T89 reference line and chlorina mutants. B) 2 μg of were loaded into SDS-Page and blotted against α-CP43 and α-Lhcb2 for T89 reference line and chlorina mutants. C) 2 μg of were loaded into SDS-Page and blotted against α-CP43 and α-Lhcb3 for T89 (aspens), Col-0 (Arabidopsis) and Tron (barley). No signal was detected in T89.


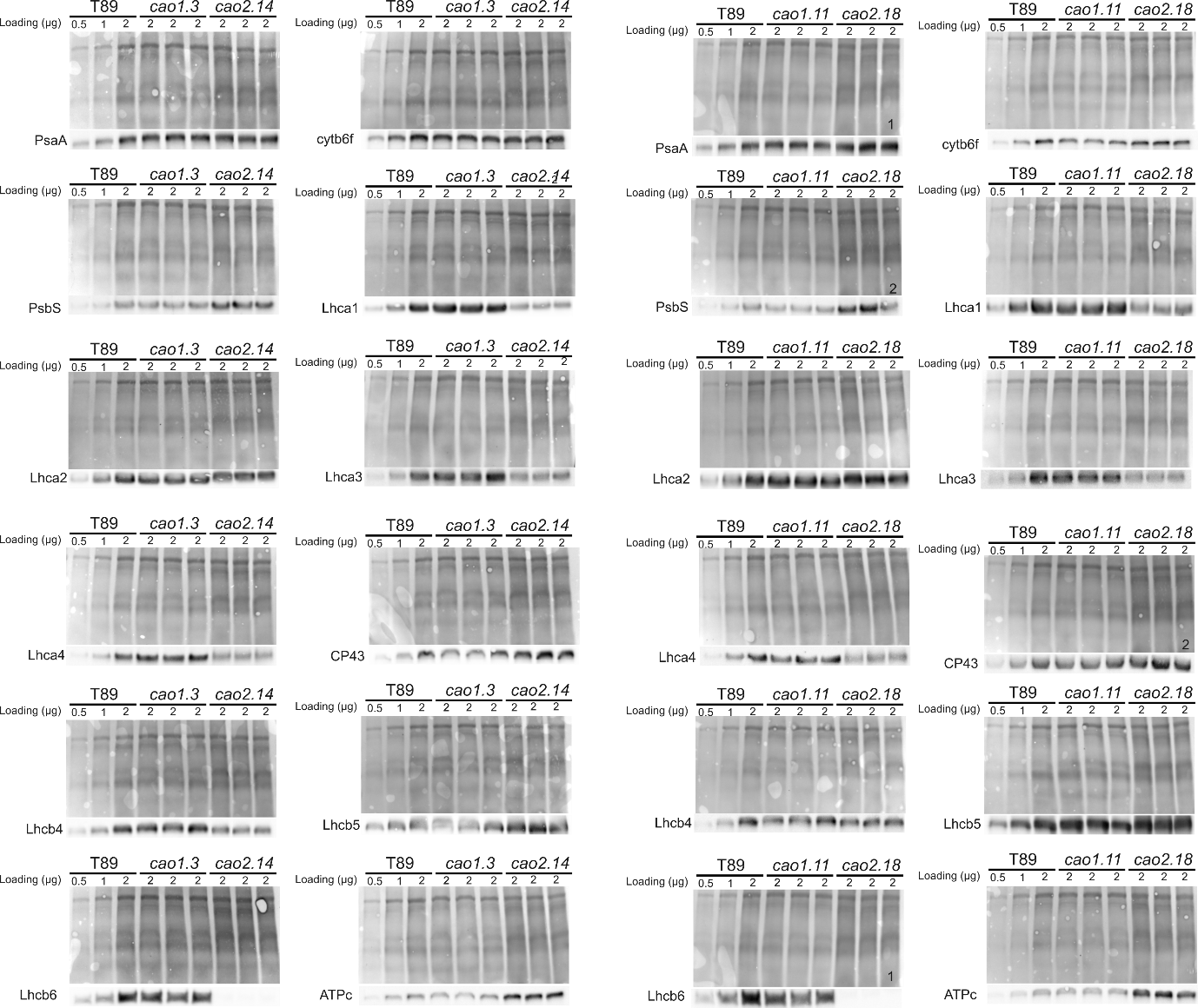


**Supplementary Figure 3. Immunoblots of core thylakoid proteins in T89 and *cao* mutants.** 0.5 to 2 μg of chlorophylls were loaded into SDS-Page and blotted against α-PsaA, α-PetA (cytb6f), α-PsbS, α-Lhca1, α-Lhca2, α-Lhca3, α-Lhca4, α-CP43, α-Lhcb4, α-Lhcb5, α-Lhcb6 and α-ATPc. Ponceaus for each blot are shown. 1 and 2 indicate that the same membrane has been used to detect different antibodies.

**
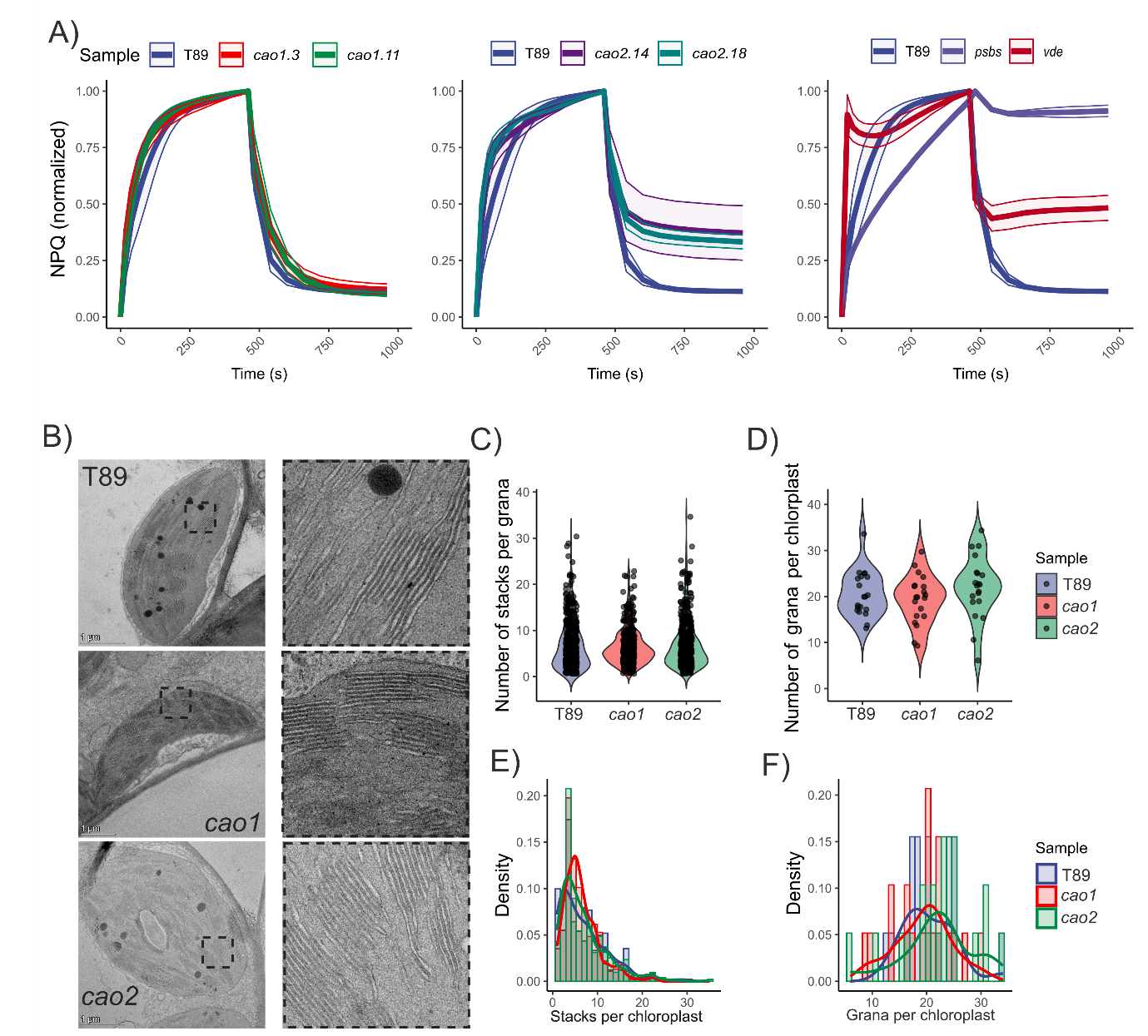
**

**Supplementary Figure 4. LHCII, PsbS and zeaxanthin modulate NPQ in aspens.** NPQ induction curves from T89, *cao1*, *cao2*, and qE mutants were normalized to maximal NPQ. For clarity, the responses of *cao1*, *cao2*, and qE mutants are shown in contrast to the T89 reference line. Notably, in *cao1* and *cao2* the early qE-dependent induction phase (20 s) is faster than in T89, whereas in *vde* is unchanged and in *psbs* is slower. The *vde* mutant shows a back reaction in NPQ during the induction phase, producing a reduction in total NPQ amplitude. In contrast, *cao1* and *cao2* exhibit a normal NPQ progression, indicating the presence of zeaxanthin formation. B) T89, *cao1.11* and *cao2.18* electron micrographs of representative chloroplasts. Total of 20 chloroplasts were counted per sample. C) Number of stacks per grana. D) Number of grana per chloroplast. E-F) Density plot showing the frequency distribution of stacks per grana and number of grana per chloroplast.


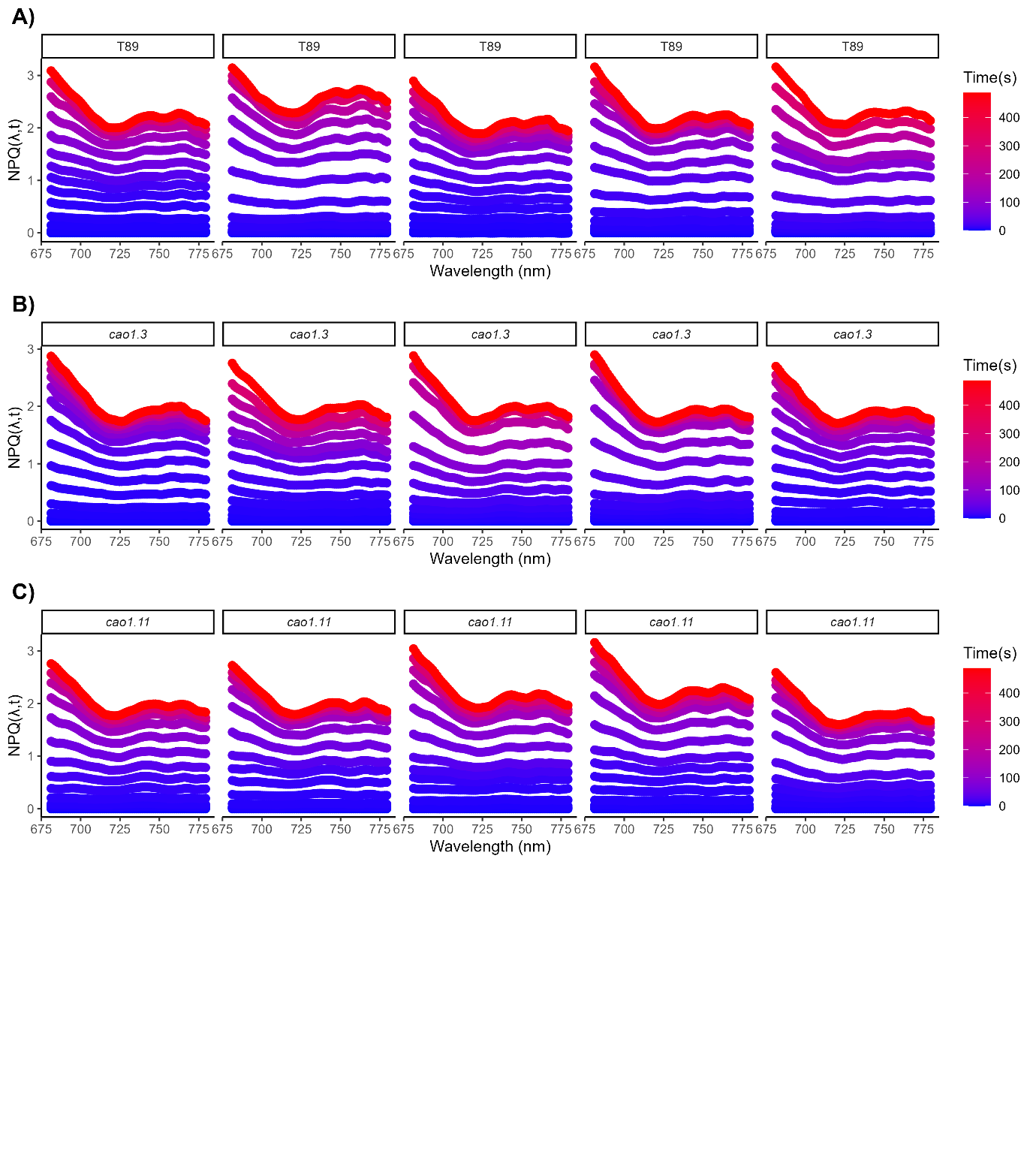


**Supplementary Figure 5. T89 and *cao1* NPQ evolution spectra.** T89 and *cao1* mutants were illuminated with 1000 μE. NPQ spectra was recorded as NPQ (λ,t)= F (λ,t_0_) / F (λ,t) - 1. A), B), C) show NPQ evolution spectra from T89, *cao1.3* and *cao1.11*, respectively. Blue spectra are associated to the initial phase of the NPQ induction, whereas red spectra indicate quenched spectra after 8 minutes of actinic light exposure. Each NPQ evolution spectra represents one biological replicates. Data is from 5 biological replicates.


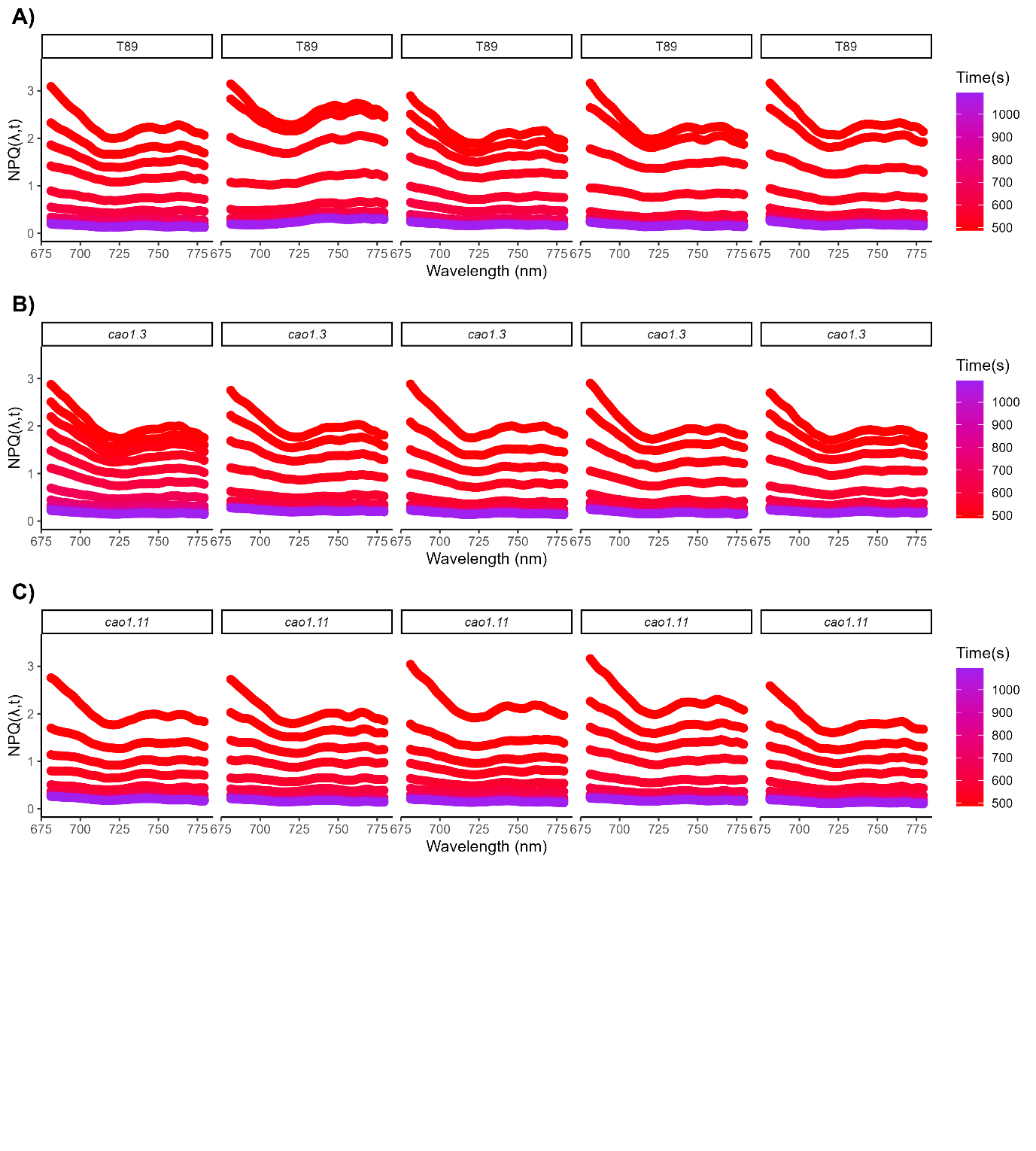


**Supplementary Figure 6. T89 and *cao1* relaxation spectra.** T89 and *cao1* mutants were illuminated with 1000 μE. NPQ spectra was recorded as NPQ (λ,t)= F (λ,t_0_) / F (λ,t) - 1. A), B), C) show NPQ relaxation evolution spectra from T89, *cao1.3* and *cao1.11*, respectively. Red spectra are associated to the end of the initial phase of the NPQ induction, whereas purple spectra indicate spectra after 10 minutes of dark relaxation. Each NPQ evolution spectra represents one biological replicates. Data is from 5 biological replicates. Note that from the top quenched spectra measured at 487.5 s, the subsequent spectra were recorded at 493.1, 503.6 and 524.2 s in dark, where NESD is almost abolished.


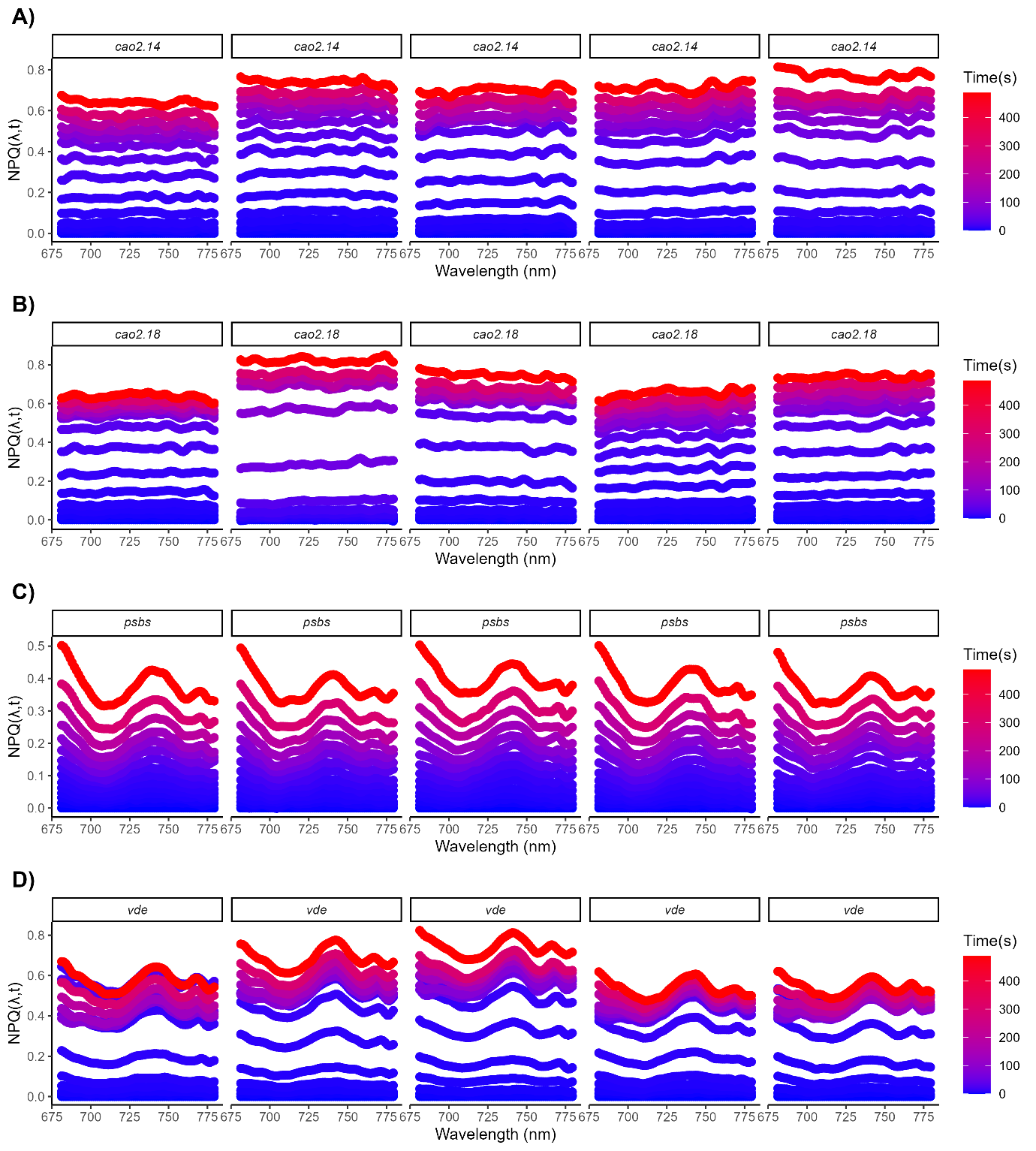


**Supplementary Figure 7. NPQ evolution spectra of NPQ mutants.** NPQ mutants, *cao2*, *psbs* and *vde* were illuminated with 1000 μE. NPQ spectra was recorded as NPQ (λ,t)= F (λ,t_0_) / F (λ,t) - 1. A), B), C) and D), show NPQ evolution spectra from *cao2.14*, *cao2.18, psbs* and *vde*, respectively. Blue spectra are associated to the initial phase of the NPQ induction, whereas red spectra indicate quenched spectra after 8 minutes of actinic light exposure. Each NPQ evolution spectra represents one biological replicates. Data is from 5 biological replicates.


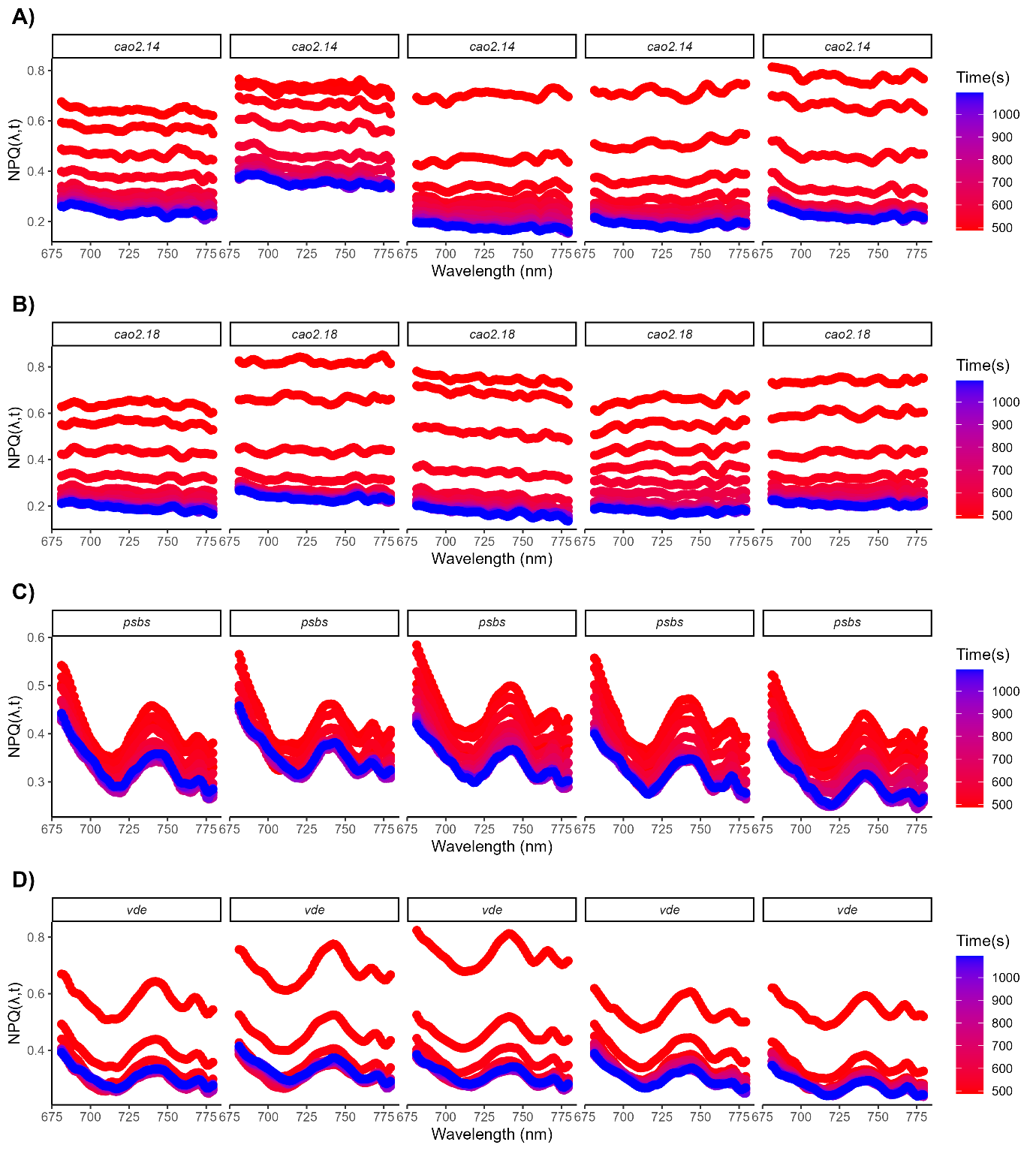


**Supplementary Figure 8. NPQ relaxation evolution spectra of NPQ mutants.** NPQ mutants, *cao2*, *psbs* and *vde* were illuminated with 1000 μE. NPQ spectra was recorded as NPQ (λ,t)= F (λ,t_0_) / F (λ,t) - 1. A), B), C) and D), show NPQ relaxation evolution spectra from *cao2.14*, *cao2.18, psbs* and *vde*, respectively. Red spectra are associated to the end of the initial phase of the NPQ induction, whereas purple spectra indicate spectra after 10 minutes of dark relaxation. Each NPQ evolution spectra represents one biological replicates. Data is from 5 biological replicates.

**Time-resolved integrated chlorophyll fluorescence vs Time-resolved spectrally resolved chlorophyll fluorescence.**

Here, we present additional analysis and explanations to clarify some of the principles underlying both methods, as well as the reasons for their discrepancies. Traditionally, time-resolved integrated chlorophyll fluorescence, or PAM fluorometry, has been widely used to monitor real-time changes in chlorophyll fluorescence at room temperature (Pfündel, 2021). The underlying assumption of this method is that changes in chlorophyll fluorescence primarily reflect alterations in PSII lifetime, which is the main contributor to variable fluorescence. That method in combination with reverse genetic, allow to the development of the NPQ paradigm which is constantly under discussion (Ruban and Saccon, 2022).

This technique relies on detecting fluorescence at a given wavelength, typically above 710 nm by using long-pass filters (Pfündel, 2021). While this approach circumvents one problem, it introduces another. PSII emission is strongest at 686 nm, yet the fluorescence signal at this wavelength is relatively weak compared to longer wavelengths because much of it is reabsorbed by the photosystems themselves. In other words, the probability of a photon escaping the leaf tissue at shorter wavelengths is low. In contrast, fluorescence detected at 710 nm or longer is less affected by reabsorption, increasing the likelihood of photon detection. The trade-off, however, is that shorter-wavelength detection emphasizes signals from the upper leaf layers, while longer-wavelength detection integrates signals from a greater number of chlorophylls across the entire tissue but loses critical details about NPQ. To avoid reabsorption effects, recent ultra fast time-resolved fluorescence studies have deliberately focused only on the upper leaf layers (Farooq et al., 2018). While effective in minimizing distortion product of chlorophyll reabsorption, this strategy risks oversimplifying NPQ dynamics and overlooking how leaves adapt to internal light gradients.

Another important aspect that has sparked considerable discussion in the field is the appearance of new emitting species during NPQ. Consequently, changes in chlorophyll fluorescence intensity do not necessarily reflect only alterations in PSII lifetime but also integrate other processes, such as the effects of the light gradient and NESD associated with NPQ sub-processes (Horton et al. 2005; Holzwarth et al. 2009; Johnson and Ruban, 2009; Farooq et al., 2018). It is therefore an oversimplification to assume that NPQ can be accurately quantified from integrated chlorophyll a fluorescence alone, since these additional properties remain hidden in integrated chlorophyll fluorometers.

By contrast, steady state spectrally time-resolved methods, as the one described in this work, provide a more detailed perspective. While based on the same fundamental principles, these techniques make it possible to evaluate, at least in part, the complexity of light gradients, the emergence of new emitting species, and NPQ itself. However, it must be acknowledged that current tools are still insufficient to fully disentangle these processes. For this reason, NPQ spectra should be regarded as emergent properties of the system rather than direct, isolated readouts. Here, we give a full description of some of the analysis that can be performed by using this technique and their implications.

The first factor, as noted above, is the influence of the light gradient within the leaf. This phenomenon is a natural consequence of light penetration, which becomes progressively attenuated with depth, generating a heterogeneous light response across the leaf tissue. Spectral deconvolution can help to disentangle this relationship between incident light and leaf optical properties. Instruments such as PAM offer the option of dual-excitation, which produces a more homogeneous light gradient but at the cost of biological relevance, since in nature it is highly unlikely that a leaf would receive the same light intensity-quality simultaneously from both sides. By contrast, systems like Licor-6800 or Joliot type spectrophotometer (JTS-100) employ directional excitation, providing a more realistic representation of natural conditions. Similarly, ChloroSpec applies a front-face excitation approach, as is commonly done in ultra fast time resolved methods (Chukhutsina et al., 2019).


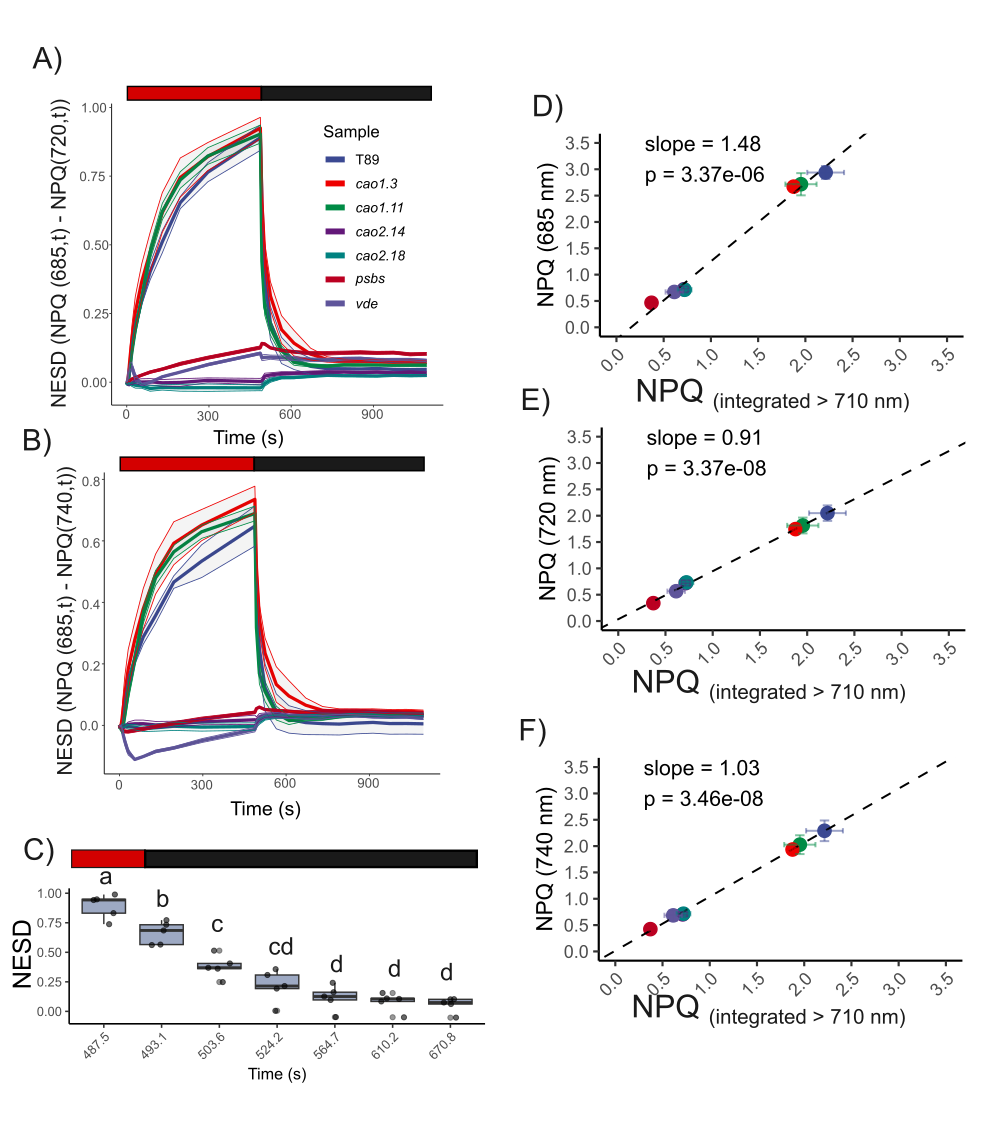


**Supplementary Figure 9.** **The complexity of NESD.** NPQ spectral inductions were performed at 1000 μmol photons m^-2^s^-1^ in collection of NPQ aspen mutants. New emitting species development (NESD) was calculated from NPQ evolution spectra as (NPQ_(685,t)_ – NPQ_(λ,t)_ ), where λ = 720 nm in A) and 740 nm in B). Data is mean ± s.d (n = 5 biologically independent experiments). C) NESD relaxation in T89 when λ = 720 nm. Shared letters indicate non-significant differences between the groups (Tukey's tests, p <0.05). In D), E) and F) Linear regression is shown for 685, 720 and 740 nm when compared to NPQ (integrated > 710 nm), respectively. NPQ integrated represent the value obtained for each NPQ spectra at any given time, integrated above 710 nm where traditional chlorophyll fluorometers detect.

This light gradient that is progressively attenuated with depth can be spectrally followed by monitoring different wavelengths (Genty et al., 1989; Pfündel, 2021). In general, detection at 686 nm will reflect changes in chlorophyll fluorescence at upper layers whereas detection at longer wavelengths will reflect overall changes with a larger effect of deeper layers. Therefore, while combining both methods some information can be extracted about the relationship between NPQ and the light gradient, as well as PSII energy partitioning. However, on top of this process there are other subprocesses that takes place in leaves, where although its origin is still a matter of debate, they have large contribution during NPQ, known as NESD (Fig. 2 and Supplementary Figure 9).

The over-simplified interpretation of NESD, which is that NPQ decreases as a function of the light gradient will imply that at longer detection wavelengths NPQ will decrease. This statement simply does not hold. For example, in T89 NESD is larger when calculated at 720 nm than at 740 nm. In simple words, NPQ is smaller at 720 nm than at 740 nm, although according to the light gradient the opposite will be expected (Supplementary Figure 9 D-F). NPQ in ref. lines usually decreases at 720 nm and increases at longer wavelengths, as can be seen in the NPQ spectra presented in this work (Supplementary Figure 3).

These is in agreement with the traditional interpretation that during NPQ, sub-processes are producing a red-shift in the spectra of PSII which end-up in a reduction of the overall amount of chlorophyll fluorescence quenching at certain specific wavelengths (Horton et al. 2005; Holzwarth et al. 2009; Johnson and Ruban, 2009; Farooq et al., 2018). Here, it should be also contemplated that during PSII quenching, PSI fluorescence becomes more relevant therefore could have a contribution at these specific wavelengths, since PSI dominates at 720 nm at RT. However, in the absence of *vde* the statement that the light gradient or PSI unmasking is producing the overall effect, is not possible. In this case, NESD has negative values when calculated at 740 nm during the induction phase, whereas is positive at 720 nm. In other words, NPQ is larger at 740 nm for *vde* than at 686 nm, challenging some of the direct interpretations about NPQ in these mutants.

Another aspect that is worth to mention, is how accurately NPQ capacity is determined by PAM integrated methods. In Supplementary Figure 10, NPQ spectra from aspen T89 and *cao2* at 1000 µE are shown. To approximate conditions of PAM fluorometry (Pfündel, 2021), NPQ values above 710 nm were integrated. The same method has been applied to obtain the values of Supplementary figure 9.

Under these conditions, the difference between the two mutants at the endpoint of NPQ induction was 2.18 versus 0.71, corresponding to a 3:1 ratio. In contrast, when evaluating values at 686 nm, which largely exclude NESD and primarily reflect PSII quenching in chloroplasts located near the leaf surface, the difference increased to 2.93 versus 0.71, yielding a ratio of 4.13. This indicates that NPQ capacity in T89 is underestimated by roughly 48% relative to *cao2* when assessed using conventional methods. This observation aligns with the model describing the physical contribution of LHCB to NPQ, in which smaller antenna systems such as *cao2* are expected to exhibit a 4–5 fold relationship compared with reference lines, assuming a PSII antenna size of 150–185 Chl per PSII RC for T89 (Nicol *et al*., 2019).


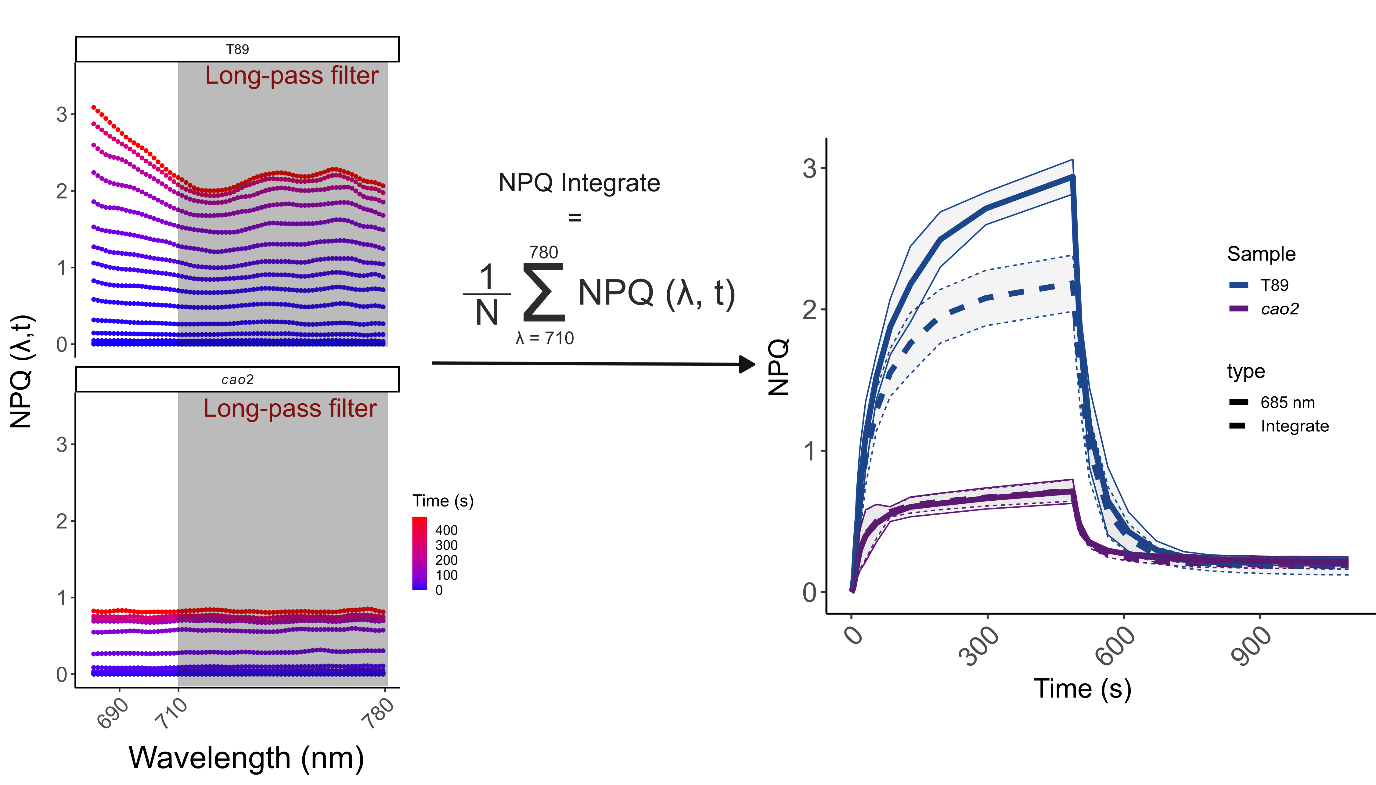


**Supplementary Figure 10.** **NESD affects the quantification of NPQ.** NPQ spectral inductions were performed at 1000 μmol photons m^-2^s^-1^ in T89 and *cao2*. NPQ spectra was integrated at any given time for 5 biological samples and used to reconstruct the NPQ integrate spectra according to the formula described. The cut-off was settled at 710 nm, as usually works for long-pass filters (Pfündel, 2021) and integrate up to 780 nm. NPQ inductions in the right compare NPQ (685,t) vs NPQ Integrate, indicated by solid or dashed lines, respectively.

In Supplementary Figure 11, we show that when NPQ induction curves are recorded from the same biological samples (different leaves of similar developmental age) using different instruments, the NPQ values integrated from Chlorospec are approximately 10% lower than those obtained with the DUAL-PAM-100. The latter has a NIR module that reflects part of the light transmitted, which reduces the light gradient effect by red actinic illumination. Therefore, a lower NPQ estimate from Chlorospec-Integrated is not unexpected. However, under natural conditions, dual excitation with the same spectral quality and, importantly, the same photon flux is highly unlikely. Consequently, eliminating the light gradient should be regarded as a trade-off between biological relevance and biophysical control of the system.

When DUAL-PAM-100 NPQ is compared specifically with Chlorospec detection at 686 nm, the integrated Chlorospec values underestimate NPQ by at least 30%. This indicates that, although reflection from NIR may partially alleviate the influence of the actinic light gradient, it is not sufficient to cancel-out the NESD effect which substantially mask the true magnitude of NPQ.


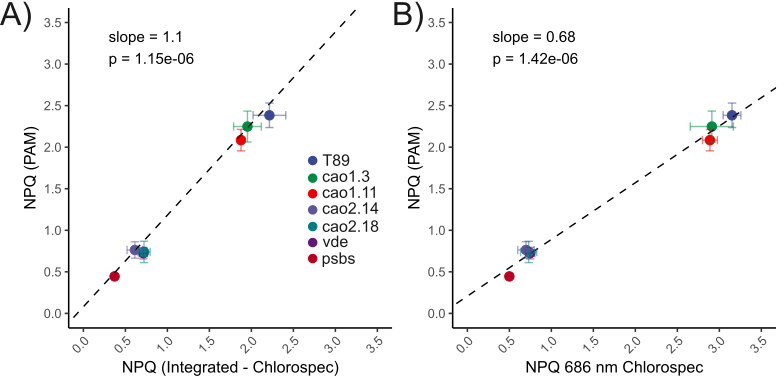


**Supplementary Figure 11.** **Light gradient influences NPQ.** A) NPQ spectral inductions (Chlorospec) and NPQ induction (DUAL-PAM-100) were performed at 1000 μmol photons m^-2^s^-1^ in T89 and npq mutant lines. NPQ spectra was integrated at 480s and compared to NPQ (PAM). Inductions were performed in same plants on randomized different leaves from save developmental stage (fully developed - positions 7 to 9) at the same day. Linear regression between variables was performed (slope). B) Same as A, but values detected at 686 nm were compared to NPQ (PAM).


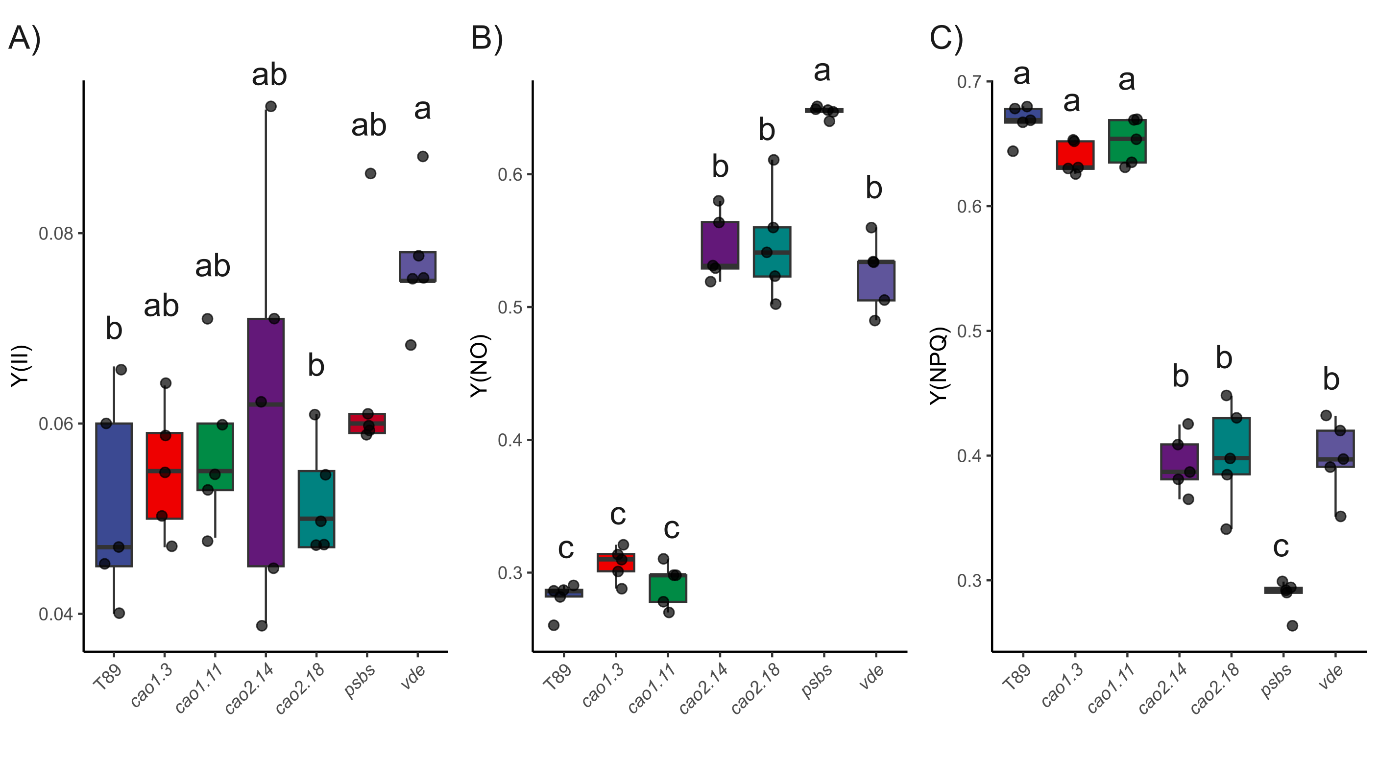


**Supplementary Figure 12. PSII energy partitioning from PAM fluorometry.** NPQ inductions were performed in aspens npq mutants and Y(II), Y(NO) and Y(NPQ) were determined in A), B and C), respectively. Data represents values achieved once T89 reached steady state at 460s of the NPQ induction. Each point indicates a different biological replicate (n = 5). Shared letters between groups indicates non-significant differences according to Tukey’s test (p < 0.05).

**
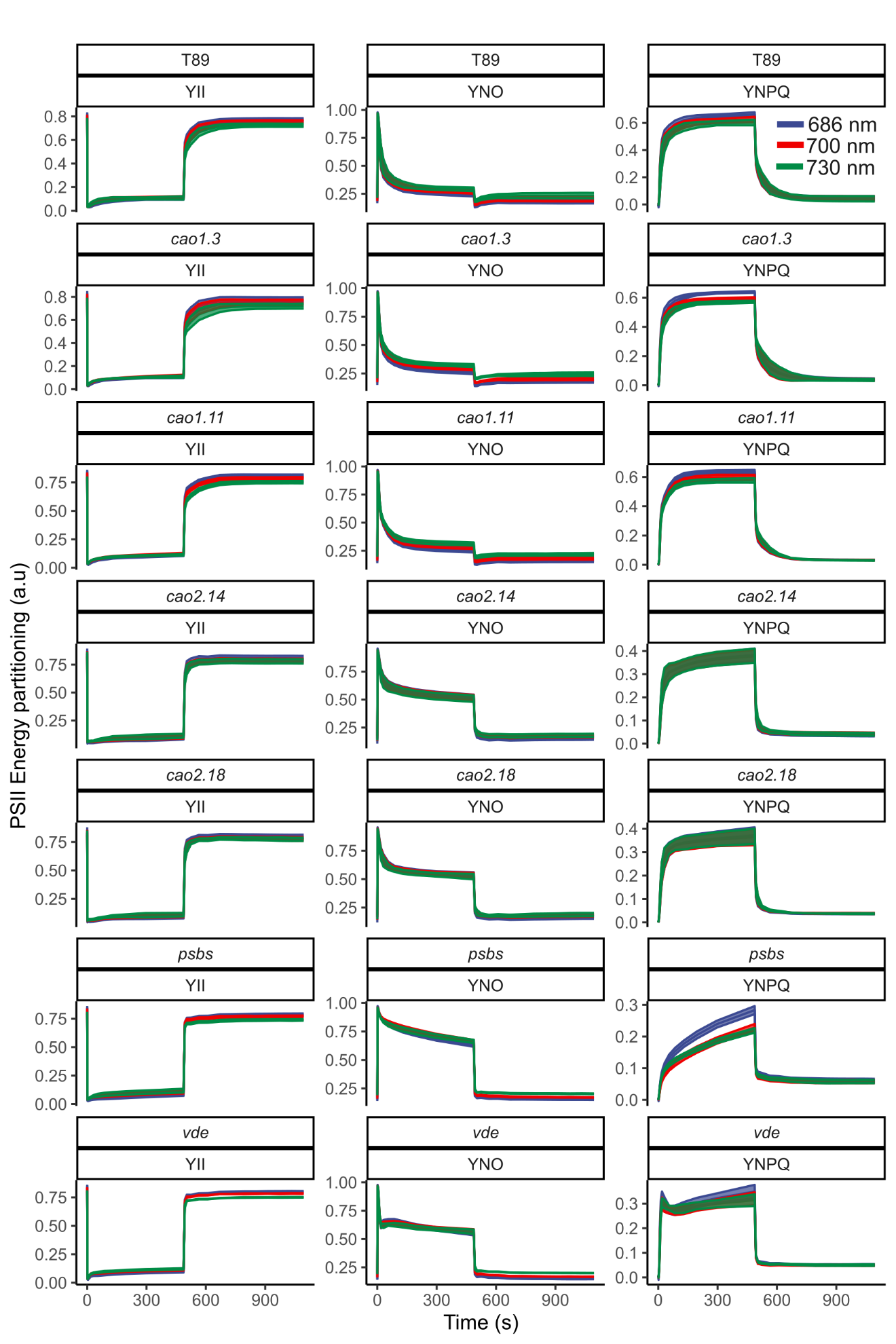
**

**Supplementary Figure 13. Aspens npq mutants PSII energy partitioning heterogeneity.** Y(II), Y(NPQ) and Y(NO) were monitored in T89 and *cao1, cao2, psbs and vde* upon NPQ inductions at 1000 μE. Each plot represents the values for genotype and the respective parameter indicated at the top of each trace. Data is mean ± s.d (n > 5 biologically independent experiments).


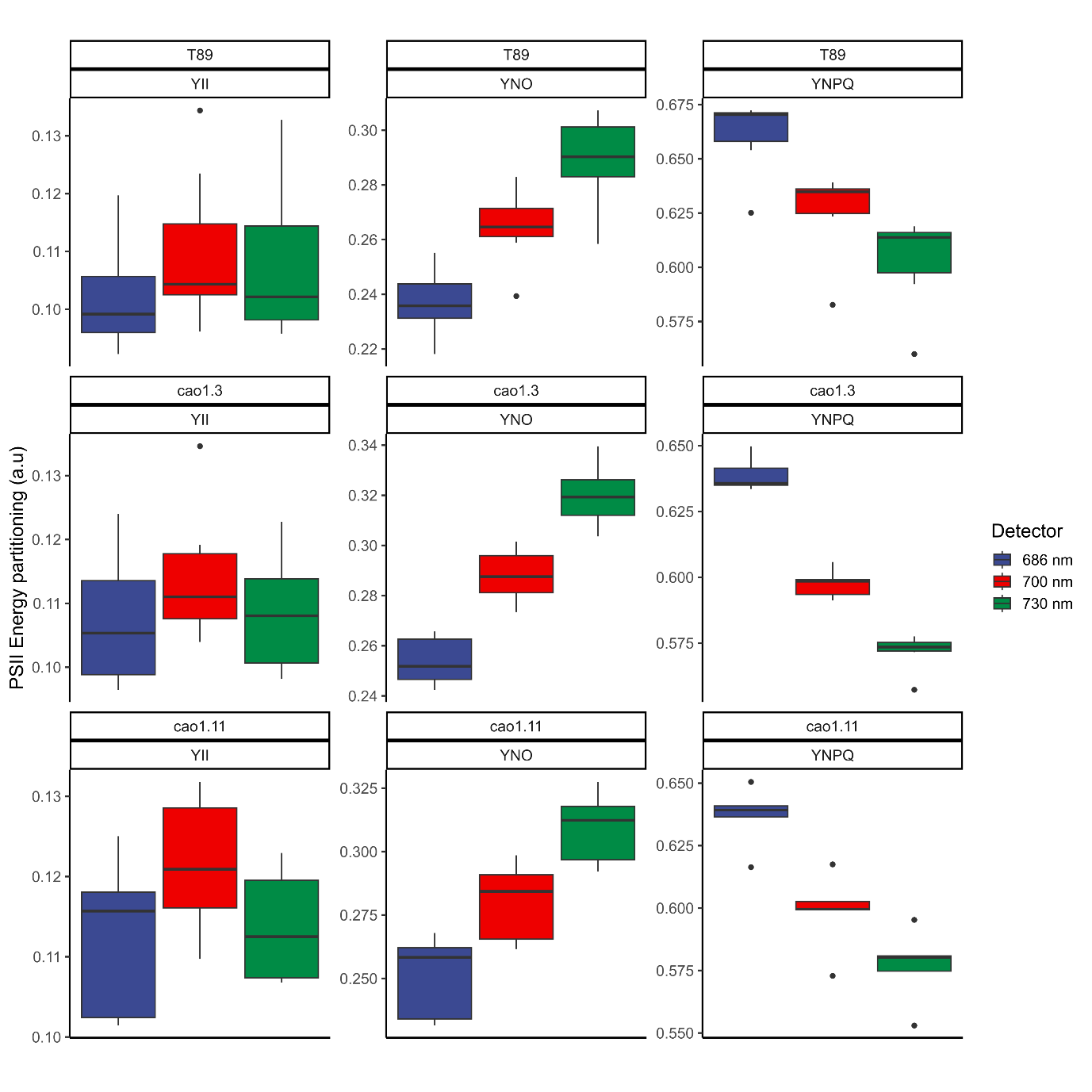


**Supplementary Figure 14. T89 and *cao1* PSII energy partitioning heterogeneity.** Y(II), Y(NPQ) and Y(NO) were monitored in T89 and *cao1* upon NPQ inductions at 1000 μE. At the top is indicated for each boxplot genotype and respective parameter for PSII energy partitioning measured at 487.5 s during the light induction phase. Data is from at least 5 independent biological replicas.


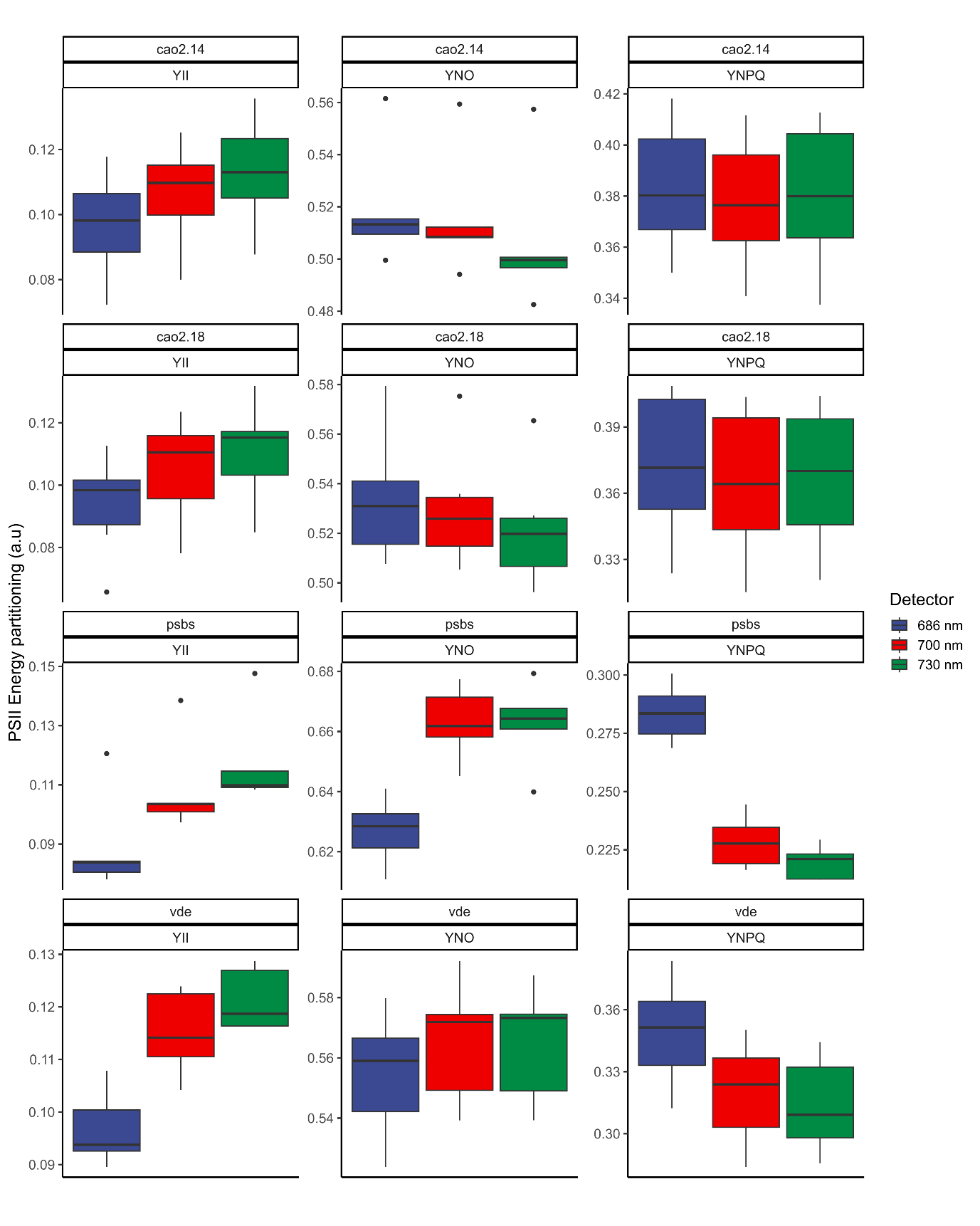


**Supplementary Figure 15. Aspen NPQ mutants PSII energy partitioning heterogeneity.** Y(II), Y(NPQ) and Y(NO) were monitored in *cao2*, *psbs* and *vde* upon NPQ inductions at 1000 μE. At the top is indicated for each boxplot genotype and respective parameter for PSII energy partitioning measured at 487.5 s during the light induction phase. Data is from at least 5 independent biological replicas.


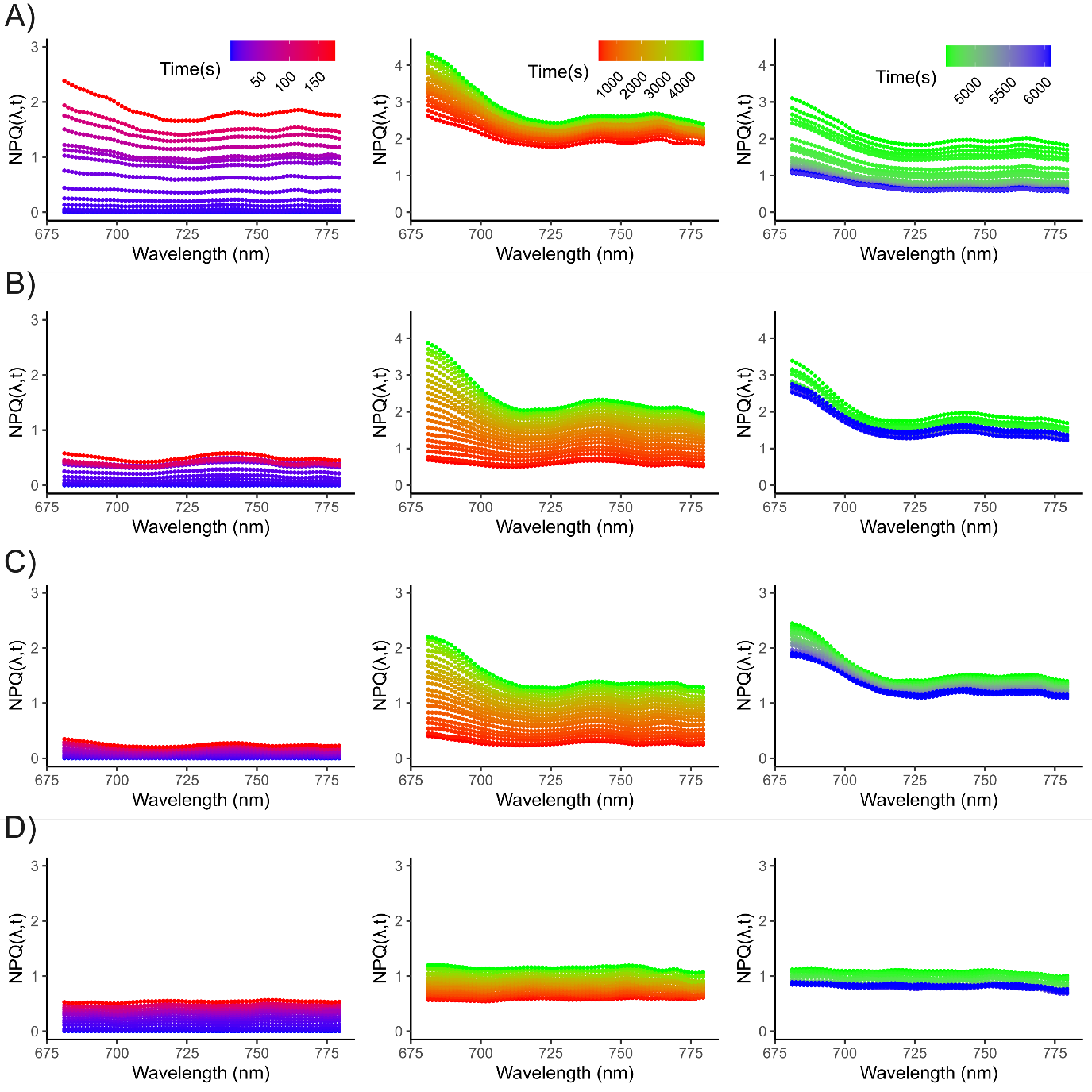


**Supplementary Figure 16. Sustained NPQ spectra of aspen npq mutants**. A), B), C), and D) show the NPQ spectra for T89, *vde*, *psbs*, and *cao2*, respectively. The left panels show the NPQ spectra during the first 200 seconds, highlighting the induction of qE and qZ. In T89, the spectra are highly heterogeneous, while the mutants show more homogeneous spectra. The middle panels show the development of NES over time, from 200s to 4600s. Notably, the spectra in *vde* and *psbs* become heterogeneous as sustained quenching develops. The right panels show the NPQ spectra during the relaxation phase. Sustained quenching is almost uniform in T89, but strongly heterogeneous in *vde* and *psbs.*

**
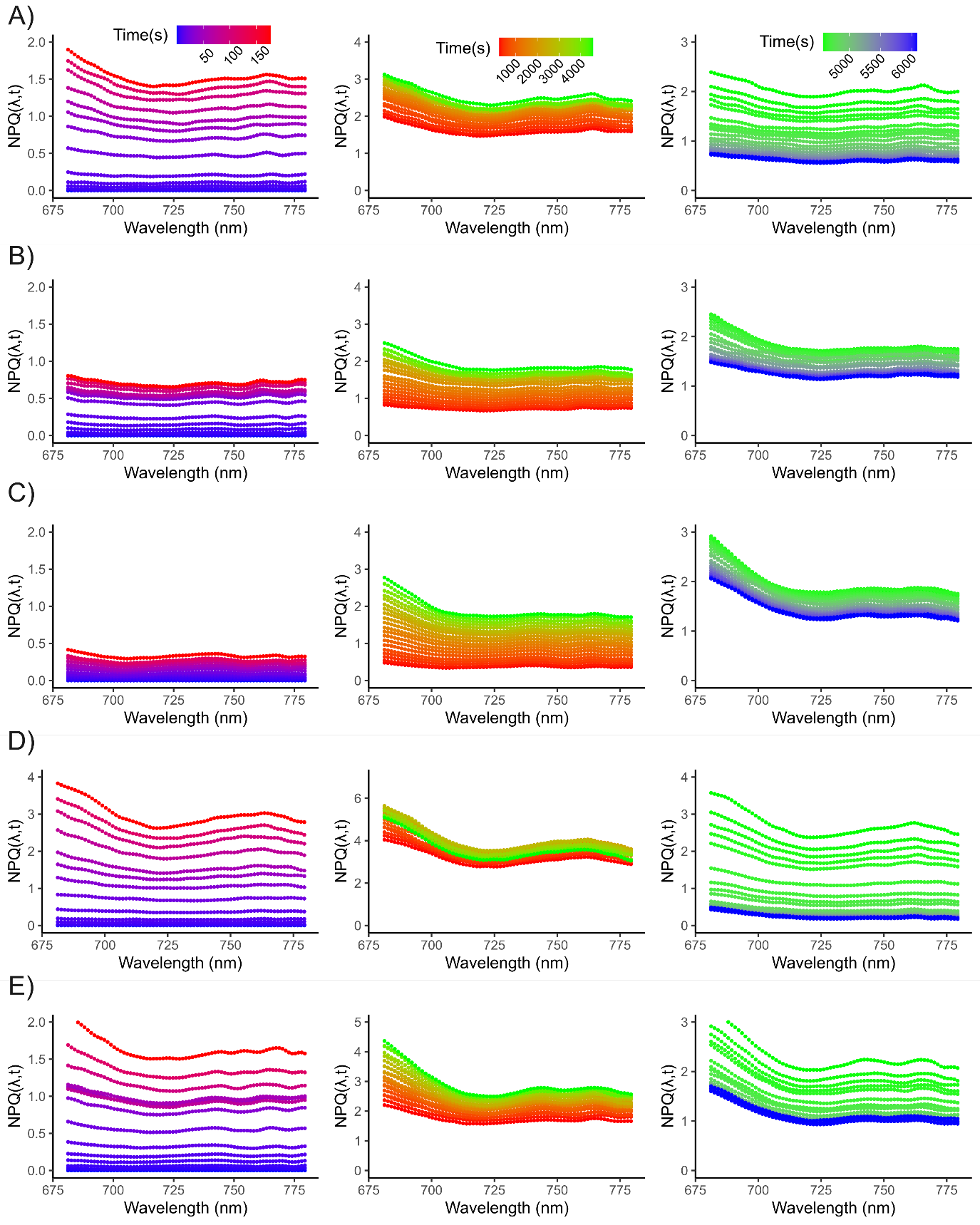
Supplementary Figure 17. Sustained NPQ spectra of *Arabidopsis* npq mutants**. A), B), C), D) and E) show the NPQ spectra for Col-0, *npq1*, *npq4*, L17 and *curt1*, respectively. The left panels show the NPQ spectra during the first 200 seconds, highlighting the induction of qE and qZ. The middle panels show the development of NES over time, from 200s to 4600s. The right panels show the NPQ spectra during the relaxation phase.


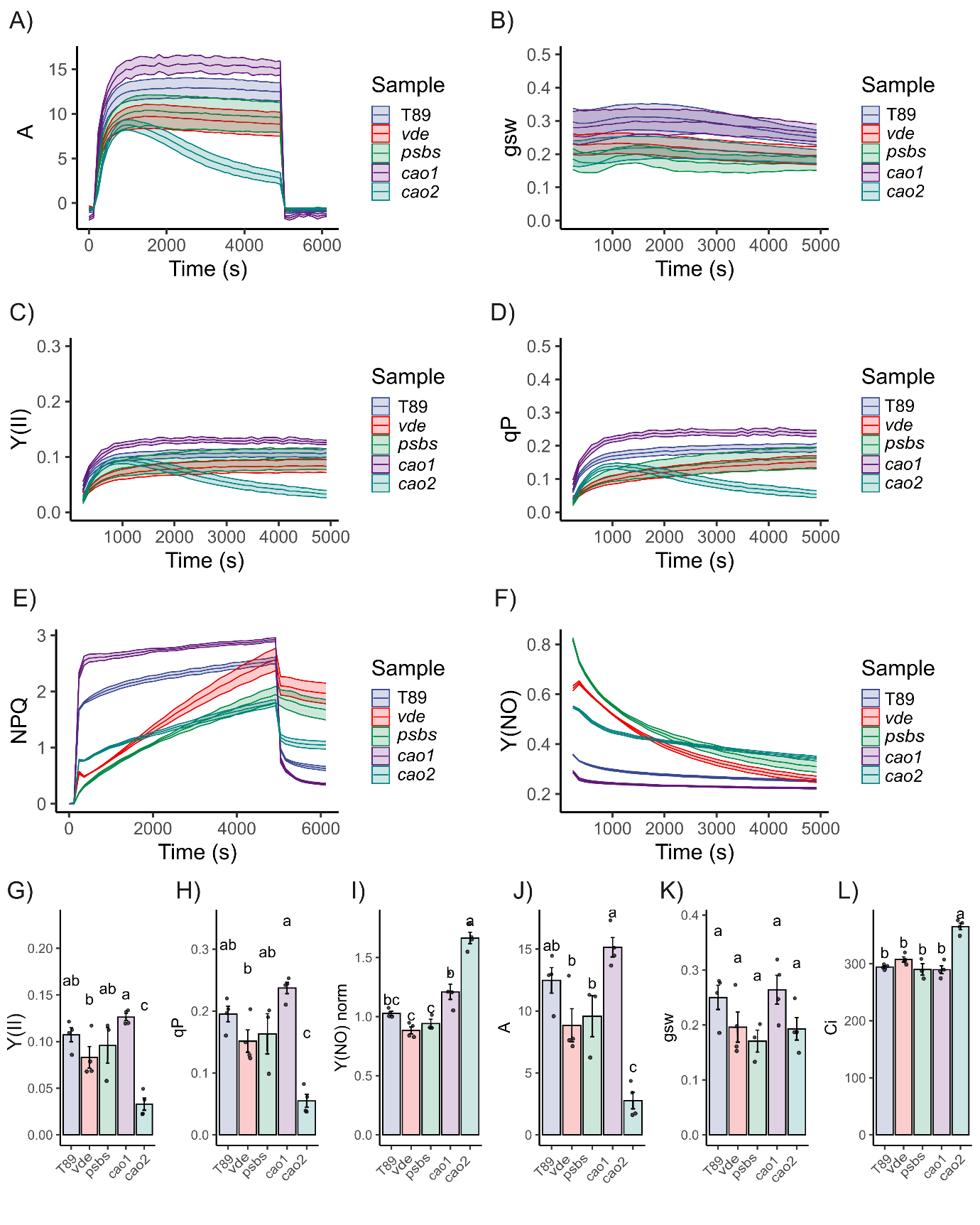


**Supplementary Figure 18. Photoinhibition and NPQ in aspen mutants after HL treatment.** NPQ kinetics were performed as described in Figure 5. A), B), C), D), E) and F) show A, stomatal conductance (gsw), Y(II), qP, NPQ and Y(NO) kinetics, respectively. Y(II) and A was normalized to their respective maximal between the range of 480s to 4920s and presented in Figure 5. Data is mean ± s.e (n > 3 biologically independent experiments). Note that *vde* at the end of the induction phase has even higher NPQ than T89 and in both *vde* and *psbs* a large amount of sustained quenching is developed which has little effect on CO_2_ assimilation. Note that the signature of high Y(NO) decreases in time in *vde* and *psbs* mutants. Although c*ao2* has a smaller absorption cross-section and lower chlorophyll content per area, assimilation declines up to 75% during HL treatments. At the relaxation phase, Y(NO) does not relax in *cao2* suggesting the presence of broken PSII RC. G), H), J), K) and L) show values at the end of the high light treatment for Y(II), qP, A, gsw and Ci, respectively. I) show values at the end of the dark-relaxation phase for Y(NO) normalized to Y(NO) at t = 0. Shared letters between groups indicates non-significant differences according to Tukey’s test (p < 0.05). Each point represents a different biological replica.


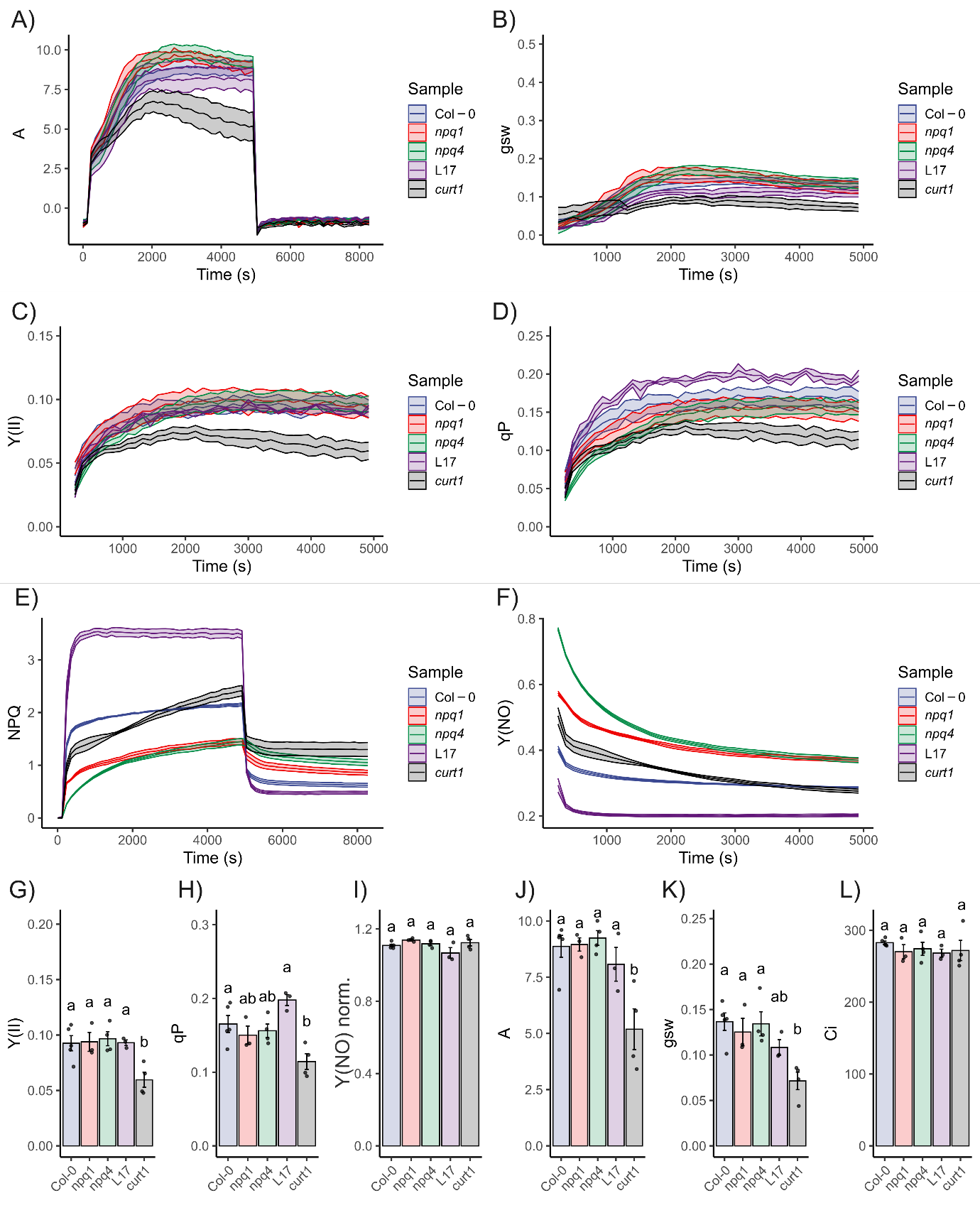


**Supplementary Figure 19. Photoinhibition is enhanced in the absence of thylakoid reorganization.** NPQ kinetics were performed at 1500 μE for 4680s with a relaxation phase of 3500s in *Arabidopsis* mutants. A), B), C), D), E) and F) show A, gsw, Y(II), qP, NPQ and Y(NO) kinetics, respectively. Y(II) and A were normalized to their respective maximum between the range of 480s to 4920s and presented in Figure 5. Data is mean ± s.e (n > 3 biologically independent experiments). Note that *npq1 and npq4* developed large amounts of sustained quenching. In *curt1* the decline in A was followed by Y(II). G), H), J), K) and L) show values at the end of the high light treatment for Y(II), qP, A, gsw and Ci, respectively. I) show values at the end of the dark relaxation interval for Y(NO) normalized to Y(NO) at t = 0. Shared letters between groups indicates non-significant differences according to Tukey’s test (p < 0.05). Each point represents a different biological replica.

**
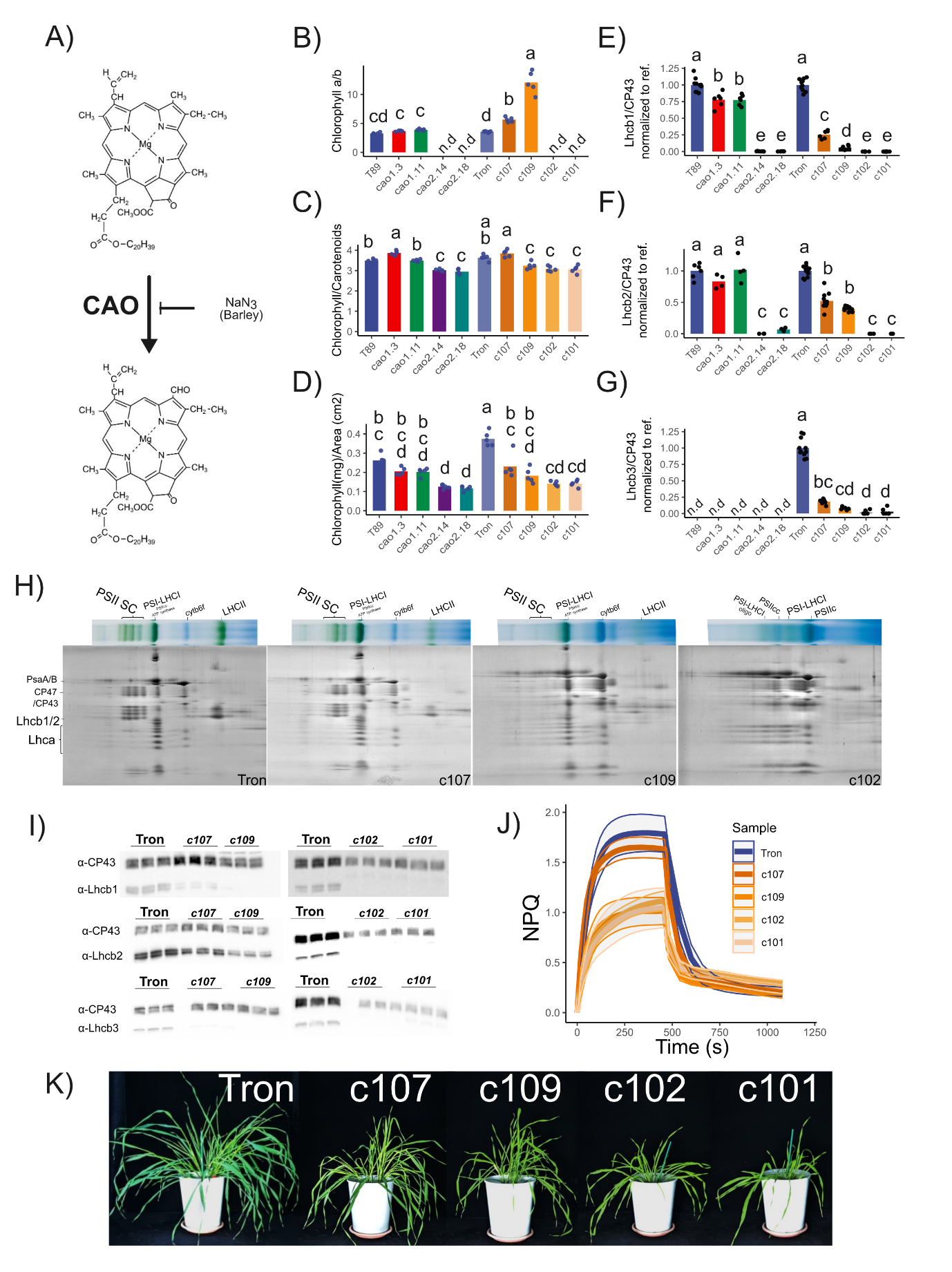
**

**Supplementary Figure 20. Chlorophyll a oxygenase activity affects PSII stability and NPQ in barley.** A) Barley cao mutants obtained by chemical mutagenesis (Bossmann et al., 1997). B-D) Pigment content of barley cao mutants compared to aspen cao mutants (Figure 1). E-G) Quantification of Lhcb1, 2 and 3 vs CP43 in barley cao mutants compared to aspen lines (Figure 1). H) BN-2D Page of barley cao mutants. Note the moderate and large reduction of LHCII in c107 and c109, respectively. The lines c102 and c101 are characterized by the absence of Lhcb proteins. I) Immunoblots with double incubation of CP43 and Lhcb1, Lhcb2 and Lhcb3. J) NPQ induction at 1000 μmol photons m^-2^s^-1^ of barley cao collection. K) Representative pictures of barley cao collection.


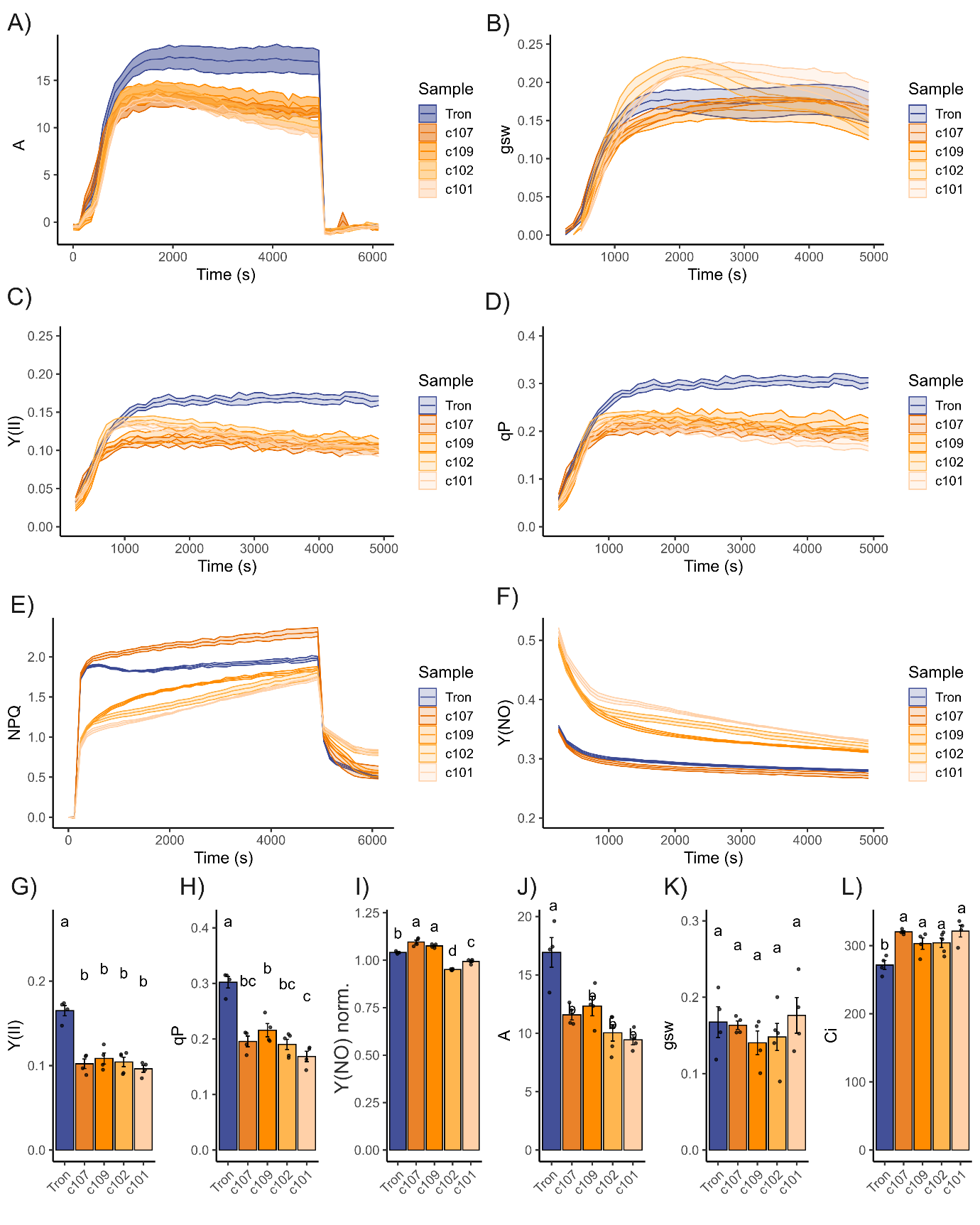


**Supplementary Figure 21. Photoinhibition is enhanced when chlorophyll a oxygenase activity is reduced in barley.** NPQ kinetics were performed at 1500 μE for 4680s with a relaxation phase of 1200s in barley cao mutants. A), B), C), D), E) and F) show A, gsw, Y(II), qP, NPQ and Y(NO) kinetics, respectively. Y(II) and A were normalized to their respective maximum between the range of 480s to 4920s and presented in Figure 5. Data is mean ± s.e (n > 4 biologically independent experiments). Note that *c101-c102* developed large amounts of sustained quenching and large decline in Y(II) and A. Here, c107 and c109 show a intermediate phenotype in agreement with their moderate decrease in PSII absorption cross-section. G), H), J), K) and L) show values at the end of the high light treatment for Y(II), qP, A, gsw and Ci, respectively. I) show values at the end of the dark relaxation interval for Y(NO) normalized to Y(NO) at t = 0. Shared letters between groups indicates non-significant differences according to Tukey’s test (p < 0.05). Each point represents a different biological replica.


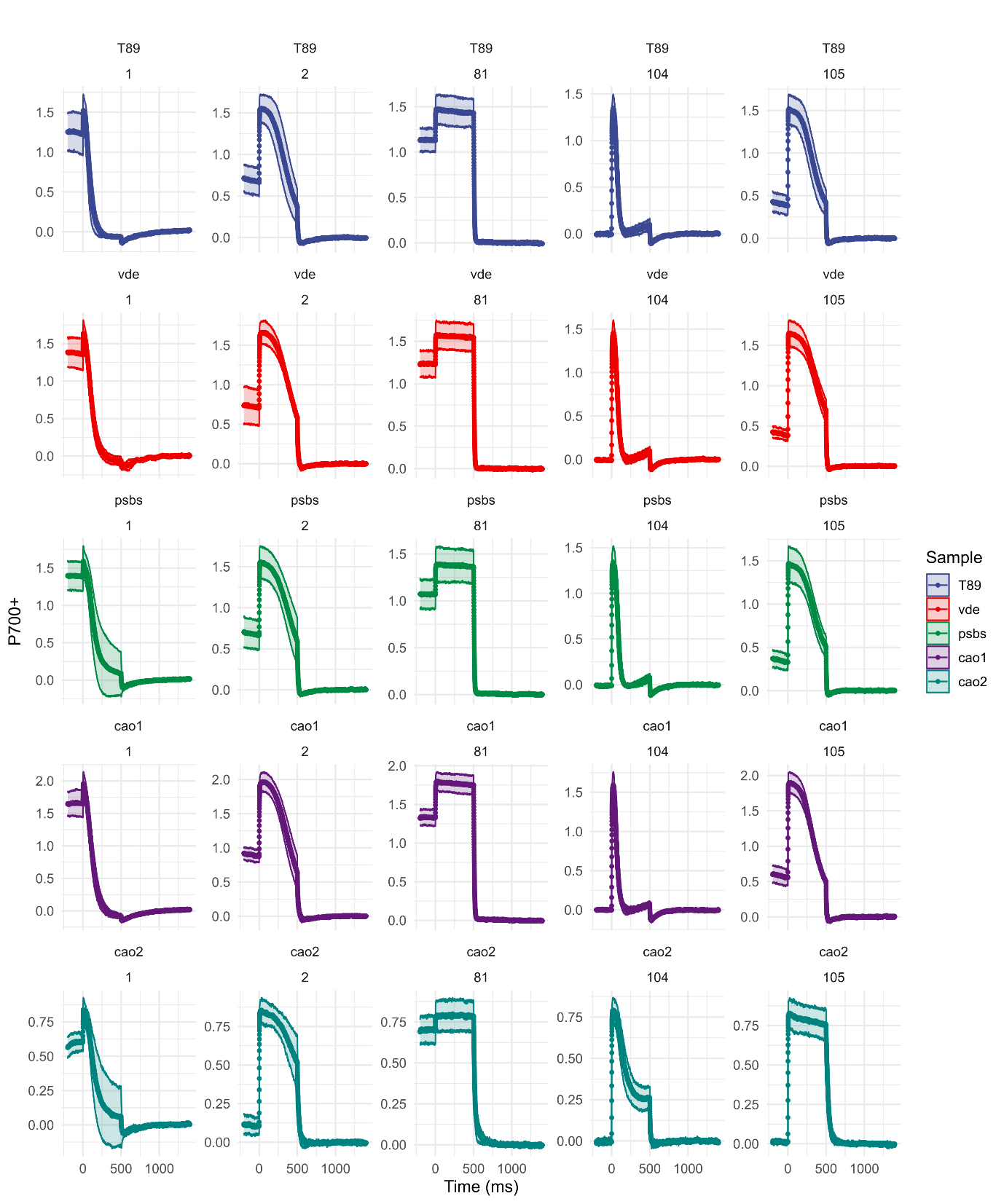


**Supplementary Figure 22. P700 pulses before and after photoinhibition in aspens npq and *cao* mutants.** NPQ kinetics were performed at 1500 μE for 4600s with a relaxation phase of 1200s in aspens *npq* and *cao* mutants. 1 shows SP (SP1) from dark-adapted plants. 2 shows SP (SP2) from dark-adapted plants after exposing them to a FRL pulse of 15 seconds. 81 shows SP from light-adapted plants after 80 minutes of high-light treatment. 104 shows SP (SP104) from the relaxation phase after 20 minutes of high-light treatment. 105 shows SP (SP105) from the phase after 20 minutes followed by a FRL pulse of 15 seconds as in 2. Although the difference between SP1 and SP2 is small, the difference between SP104 and SP105 after the photoinhibitory treatment increases to about 20%. In contrast, in *cao2* the max P700⁺ signal is identical in SP104 and SP105.


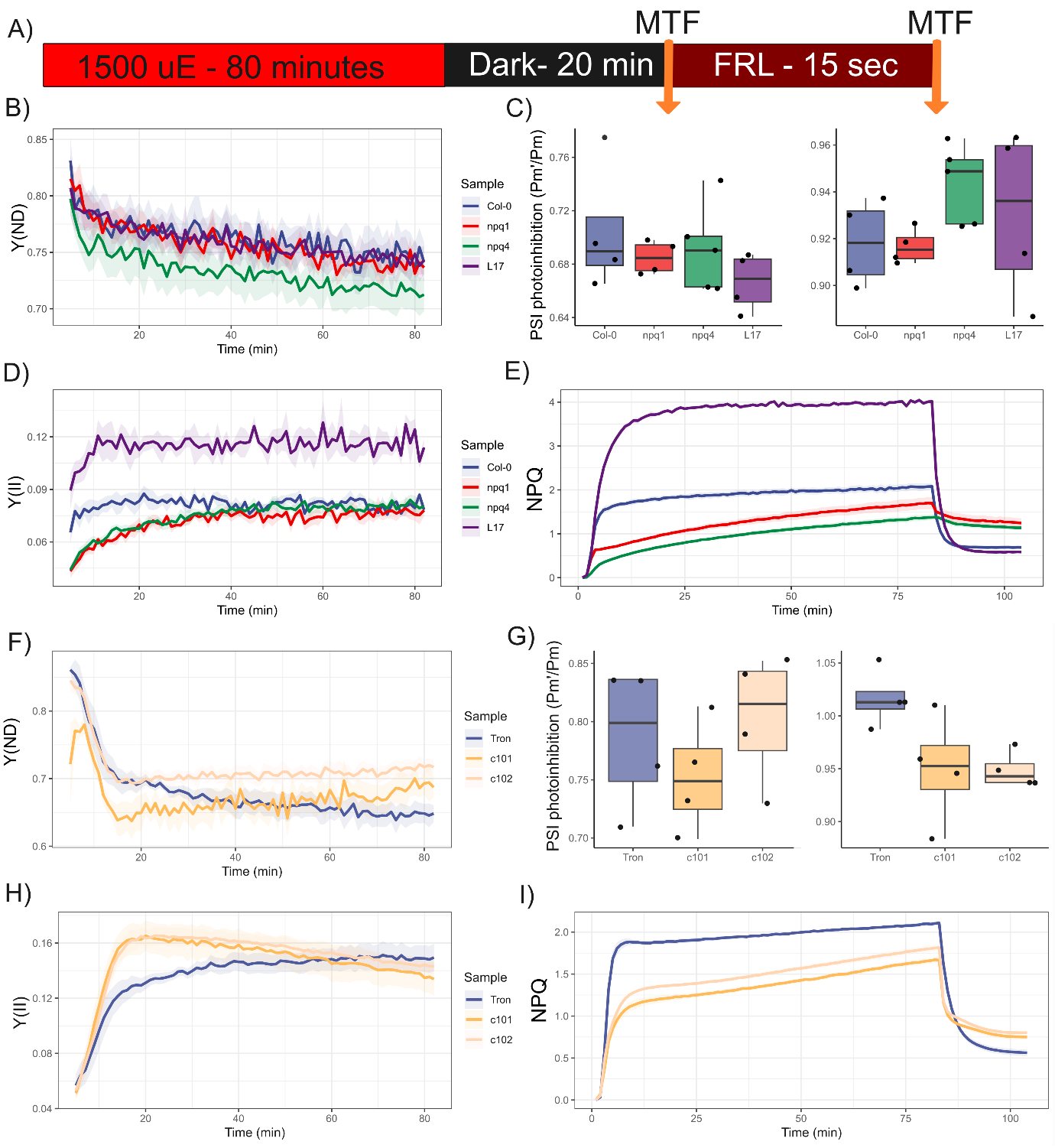


**Supplementary Figure 23. PSI photoinhibition in Arabidopsis and Barley mutants.** A Diagram of Arabidopsis NPQ inductions by integrated fluorometry and P700 pulse method (Dual-PAM-100) performed at 1500 μmol photons m^-2^s^-1^ for 4800 s followed of a relaxation phase of 1200s. At the end of the kinetic, a far-red light pulse (250 μmol photons m^-2^s^-1^ ) of 15 seconds was applied to fully oxidized P700. B-D) Y(ND) and Y(II) kinetics for Arabidopsis npq mutants during the high light treatment. Note the positive progression of Y(II) in the absence of qE during the high-light treatment*.* C) PSI photoinhibition was measured after 20 minutes of dark-relaxation by the P700 pulse method (MTF) and after a FRL pulse followed by a MTF. E) NPQ kinetics. Data is mean ± s.d (n > 4 biologically independent experiments). E-I) NPQ induction in Tron, *c101* and *c102*. Note the positive progression of Y(ND) and the decline of Y(II) in the absence LHCII (c101-c102) during the high-light treatment. Data is mean ± s.d (n = 4 biologically independent experiments).


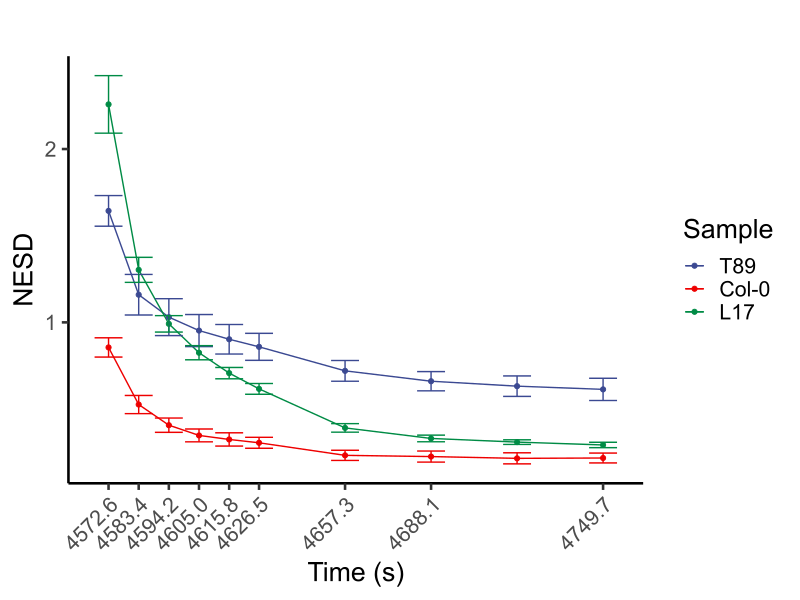


**Supplementary Figure 24. Sustained NES after photoinhibition.** NESD relaxation phase of NPQ kinetics for T89, Col-0 and L17 after HL treatment (1500 μE for 4572.6 s). Note that in Col-0 NES relax after 100 s whereas in L17 takes up to 200 s. In T89, NES are found after 200 s of dark relaxation. Data is mean ± s.d (n > 3 biologically independent experiments).
